# Polyphenol Extraction from Food (by) Products by Pulsed Electric Field: A Review

**DOI:** 10.3390/ijms242115914

**Published:** 2023-11-02

**Authors:** Vassilis Athanasiadis, Theodoros Chatzimitakos, Konstantina Kotsou, Dimitrios Kalompatsios, Eleni Bozinou, Stavros I. Lalas

**Affiliations:** Department of Food Science & Nutrition, University of Thessaly, Terma N. Temponera str., 43100 Karditsa, Greece; vaathanasiadis@uth.gr (V.A.); kkotsou@agr.uth.gr (K.K.); dkalompatsios@uth.gr (D.K.); empozinou@uth.gr (E.B.); slalas@uth.gr (S.I.L.)

**Keywords:** polyphenols, flavonoids, antioxidants, PEF, non-thermal technique, green extraction, electroporation, fresh food products, food by-products

## Abstract

Nowadays, more and more researchers engage in studies regarding the extraction of bioactive compounds from natural sources. To this end, plenty of studies have been published on this topic, with the interest in the field growing exponentially. One major aim of such studies is to maximize the extraction yield and, simultaneously, to use procedures that adhere to the principles of green chemistry, as much as possible. It was not until recently that pulsed electric field (PEF) technology has been put to good use to achieve this goal. This new technique exhibits many advantages, compared to other techniques, and they have successfully been reaped for the production of extracts with enhanced concentrations in bioactive compounds. In this advancing field of research, a good understanding of the existing literature is mandatory to develop more advanced concepts in the future. The aim of this review is to provide a thorough discussion of the most important applications of PEF for the enhancement of polyphenols extraction from fresh food products and by-products, as well as to discuss the current limitations and the prospects of the field.

## 1. Introduction

**Figure 1:**
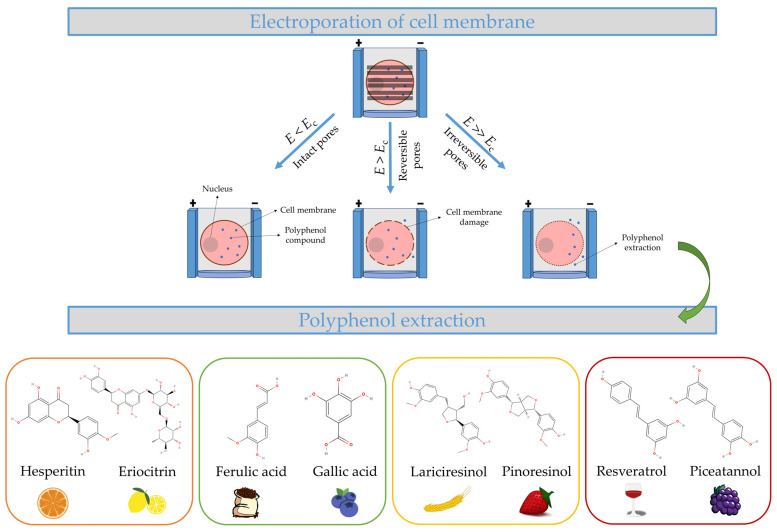
Electroporated cell membrane with bioactive compounds within several electric field strengths. Impact of critical electric field strength in the cell membrane. The four different types of polyphenols found in plant-based foods are transferred from the cell to the extraction solvent.

Polyphenols are naturally present in plant-based foods and show an extensive variety of complicated chemical structures [1,2,3]. They are composed of a phenolic ring which serves as the fundamental monomer [4]. The primary classes of polyphenols include phenolic acids, flavonoids, stilbenes, and lignans [5]. Flavonoids consist of flavones, flavonols, flavanones, isoflavones, flavanols, and anthocyanins, whereas hydroxybenzoic and hydroxycinnamic acids are types of phenolic acids [6]. These compounds are present in the human diet and are mainly derived from plant sources, including fruits, vegetables, grains, and coffee [7]. Polyphenols are a wide array of bioactive compounds that naturally occur in food sources derived from plants [8]. They are recognized for their potential as preventive agents against chronic illnesses, such as cardiovascular diseases and diabetes [9]. The four major types of polyphenols are phenolic acids, flavonoids, stilbenes, and lignans [10,11]. A visual representation of these compounds is illustrated in Figure 1. Flavonoids are highly prevalent in the context of dietary intake [12,13]. Catechin is found in a variety of fruits and beverages, most notably in tea [14]. Citrus fruits are known for their high content of hesperidin [15], whereas red fruits and berries are primarily characterized by their cyanidin content [16]. Fruits, such as apples, are known to possess proanthocyanidins and quercetin [17]. Proanthocyanidins are also present in grapes and cocoa [18], while quercetin can be found in onions and tea [19]. Finally, it should be noted that the soybean plant is primarily characterized by the presence of daidzein [20]. Polyphenolic compounds, such as lignans, are predominantly found in grains and flaxseed [21]. Due to the significant impact of polyphenols on human health, a multitude of research studies have been conducted to examine the physiological effects displayed by these compounds [22,23,24,25]. The consumption of foods rich in polyphenols has been demonstrated to contribute to a reduction in the incidence of several health conditions, such as colon cancer, liver disorders [26], cardiovascular diseases [27], and obesity [28].

The investigation of diverse conventional methodologies has been conducted to extract bioactive compounds from specific fresh food products or food waste materials [29,30,31]. Common extraction techniques include soaking, maceration, infusion, percolation, and Soxhlet extraction [32]. The efficacy of these techniques is influenced by various factors, including the choice of solvent, the solvent’s solvation capacity, the degree of agitation, and the temperature [29]. Traditional extraction methods are associated with several challenges, including extended processing durations, reduced extraction efficiencies, excessive solvent usage, possible degradation of thermolabile bioactive compounds, and the utilization of hazardous chemicals [32,33]. A selective extraction is not adequately served by conventional extraction techniques [34,35]. Furthermore, the extracted products, such as proteins and polysaccharides, may not be of high quality if these methods are used [36]. Pulsed electric field (PEF) is a processing method that implies a higher polyphenol extraction [37]. The PEF treatment is a non-thermal technique employed for food preservation, which involves the application of short bursts of electrical power to inactivate microorganisms while minimizing any detrimental impact on the food’s quality [38]. This implies that the PEF treatment aspires to enhance the accessibility of consumer-grade, polyphenol-abundant food products of superior quality [39].

The use of PEF to diffuse, osmose, press, and dry food waste and by-products has gained popularity [40]. It reduces the negative effects of regular heating methods [41,42]. Since PEF can electroporate cell membranes, it is also used as a pretreatment to boost recoveries of bioactive compounds, such as polyphenols, carotenoids, and proteins [43,44]. When applied to water, the PEF technique exhibited lower temperatures, lower solvent consumption, and improved constituent extraction rates [45]. The extraction yield could be increased with reduced energy costs, and heat-sensitive substances could be preserved; all of these by also incorporating a “green” extraction method [46,47,48]. The PEF method exhibits greater environmental sustainability and economic efficiency due to its reduced overall energy consumption and lower energy requirements per unit of processed product [49]. For this reason, various sectors of the food industry have experimented with PEF-based maceration over the past decade [50]. Apart from fresh fruits and vegetables, biomass waste generated in the agricultural and food industries is increasingly seen as a valuable bioresource that can be converted into useful products. Large quantities of wastes, including processing residues, are generated as a result of agro-industrial activity [51].

In the past few years, there has been a growing global interest among researchers in the field of processing through PEF [52,53]. Ongoing research is being conducted to investigate the impact of PEF processing on the nutritional characteristics of various food products, including fruits and vegetables [54,55,56]. By conducting an extensive literature review, our aim was to shed light on the potential impact of innovative technologies, such as PEF, in several products of the food industry. The main focus of the current study on PEF-assisted extraction was the investigation of polyphenols obtained from fresh foods as well as their by-products. Specifically, the implementation of PEF and how they could effectively enhance sustainable practices by optimizing the extraction of polyphenols was explored. Given the pressing necessity for long-term strategies aimed at enhanced global food production and waste management, this review aims to provide a comprehensive perspective on the functionality of PEF technology. Furthermore, in this review, the existing challenges and potential opportunities within this particular field were also analyzed.

## 2. Review Methodology

The study utilized three electronic databases, i.e., Google Scholar, Scopus, and Science Direct, to conduct a comprehensive search for research studies associated with the recovery of polyphenols from fresh fruits, vegetables, plants, crops, algae, as well as their byproducts, using PEF technology. The following terms were used to find articles published between 2012 and 2023 that met the review’s criteria: (“polyphenols” OR “fruit” OR “vegetable” OR “plants” OR “wheat” OR “cereal” OR “cereal grains” OR “algae” OR “food byproducts” OR “antioxidant activity” AND “Pulsed Electric Field” OR “PEF” AND “polyphenols” OR polyphenolic compounds AND “extraction” OR “isolation”).

## 3. The Impact of PEF Parameters

The most important factor in PEF is electric field strength [57]. In most cases, a stronger electric field will result in a greater amount of electroporation and cell substance transfer [58]. The extraction efficiency is measured by employing the electric field strength, which affects the targeted molecule’s surface tension, diffusivity, solubility, and viscosity [59]. In the PEF treatment, electrical impulses with a large voltage amplitude are applied. Items placed inside the chamber are exposed to electrical pulses with peak voltages of up to 80 kV/cm and durations of only some μs [60]. Depending on the effects desired and the characteristics of the food being processed, the process conditions can be altered, such as the electric field strength, pulse frequency, pulse width, shape of the pulse wave, and exposure time (which is affected by the flow rate and volume of fluid in the electrode chamber) [61].

The effectiveness of cell disintegration in releasing intracellular substances is significantly impacted by the pulse width. The membrane’s disruption is proportional to the frequency and pulse width of the applied high-voltage pulses [62]. The duration of exposure to electrical field strength for food samples is between 100 μs and 10 ms, which is sufficient to damage plant tissue at an electric field intensity of 0.5–5.0 kV/cm. The flow/speed of the food sample, along with pulse width and pulse frequency, all have an important part in calculating the total treatment time [63,64]. The specific energy of a treatment also depends on the strength of the electrical field strength, the duration of the treatment, and the electrical resistance of the treatment chamber. The treatment zone’s dimensions and configuration, as well as the conductivity of the sample food, are used to regulate the resistance [65,66].

The extraction of PEF is significantly impacted by temperature. The PEF extraction method does not require high temperatures and can therefore typically be carried out at room temperature. The extraction process is negatively impacted by temperatures above 90 °C because the viscosity of solvents decreases [67].

## 4. The Principle of Electroporation

Electroporation is a process where cells become more permeable to outside substances after being subjected to PEF through the application of brief, powerful electric pulses [68]. Electroporation precise mechanism determination remains a challenging task [69]. Attempts to explain the mechanism of reversible electroporation and electrical membrane breakdown have been proposed using experiments performed on hypothetical structures [70,71]. As a result of this imposing process at the membrane interfaces, the membrane potential rises when a biological cell is subjected to an external electric field. In Figure 1, a fundamental situation of a spherical biological cell is illustrated. The emergence of reversible or irreversible pore space in the membrane can be induced by exceeding a critical value (*E*_c_) of the electric field strength [72]. Permeabilization of the cell membrane can be either temporary or permanent, depending on the strength of the external electric fields and the frequency of the pulses [73]. Cell membranes undergo reversible permeabilization when the applied electric field is below the critical value, or when only a small number of pulses are applied [74]. However, irreversible electroporation occurs with stronger PEF treatment, leading to cell membrane disruption and cell death [75]. The process of cell disintegration is commonly acknowledged as a direct method for examining the structural characteristics of samples that have undergone different processing techniques. The cell disintegration index (*Z*_p_) is a metric used to measure the proportion of permeabilized cells in plant tissues by examining the frequency-dependent conductivity of both intact and permeabilized cells. The numerical value of *Z*_p_ varies between zero, representing intact tissues, and one, indicating tissues in which all cells have undergone permeabilization [76].

## 5. Applications of PEF in Fresh Food Products and By-Products

### 5.1. Fruits

#### 5.1.1. Prunus Fruits

The extraction of polyphenol-rich compounds from defatted apricot (*Prunus armeniaca*) kernel biomass was studied by Makrygiannis et al. [77]. To improve extraction efficiency, the use of deep eutectic solvents (DES) and PEF integration were investigated. The samples were subjected to PEF treatment for 15 min at an electric field strength of 1 kV/cm. The pulses had a frequency of 1000 μs and lasted for 10 μs each. Samples were pretreated with PEF for 15 min, and extracted for 3 h at 60 °C. The defatted apricot kernel biomass was stirred with water or DES (glycerol: choline chloride 2:1 *w*/*w*) for 15 min. According to the findings, a boost of 88% in total polyphenol content (TPC) was achieved by applying PEF before the extraction process. In a similar way, DES employment led to roughly a 70% improvement in TPC. When the two methods were combined, a 173% increase (12 mg gallic acid equivalent (GAE)/g dw) was observed. The best method for extracting bioactive compounds from defatted apricot kernels was found to be DES with PEF prior to extraction. The levels of total flavonoid content (TFC), ferric-reducing antioxidant power (FRAP), and antioxidant activity (AA) all followed a similar trend. As a result of using the aforementioned values, values for TFC, FRAP, and AA were 10 mg rutin equivalent (RtE)/g dw, 18 mol ascorbic acid equivalent (AAE)/g dw, and 12 mol AAE/g dw, respectively. When compared to water-only control extraction, the respective increases were 150%, 80%, and 71%. This research showed that bioactive compound extraction could be improved by using low-voltage PEF treatment in conjunction with a variety of green solvents, including DES.

Recently, *Prunus spinosa*, commonly known as blackthorn or sloe, possesses numerous health benefits attributed to the antioxidant and antibacterial properties present in the fruit. The research by Kotsou et al. [78] aimed to investigate the impact of different extraction parameters, such as extraction time, temperature, and solvent, on the extractability of polyphenols in *P. spinosa* fruit through US and PEF treatments along with conventional extraction (stirring). The electric field strength was set at 1.0 kV/cm with a pulse period of 1 ms, and 10 μs pulse length. In order to enhance the parameters and evaluate their impact on the antioxidant properties of the extracts, a response surface methodology was employed. The results showed that PEF treatment along with US and stirring was the most efficient way to recover a high quantity of total polyphenols. TPC was predicted by response surface methodology at 23.5 mg GAE/g dw. PEF was observed to have little to no impact on neochlorogenic acid and total anthocyanins recovery, where sole stirring and US with stirring were more efficient, respectively. However, partial least square analysis revealed that the combination of PEF along with US and stirring, with extraction for 30 min utilizing 25% *v*/*v* aqueous ethanol as solvent at 80 °C, the polyphenol extraction was enhanced. Indeed, the experimental values showed that TPC was measured at 30.74 mg GAE/g, neochlorogenic acid at 4.13 mg/g, and total anthocyanins at 125 μg cyanidin equivalent (CyE)/g. TPC was increased by 27% from a sole stirring extraction extract and by 57% than US with stirring extract. This research provides important new information about how to optimize the extraction process and how *P. spinosa* fruit could be used in the future of food science and medicine.

Cherry (*Prunus avium* L.) is one of the widespread fruits owing to its sweet taste and intense color associated with anthocyanins [79,80]. In addition to bolstering the immune system and protecting against cancer, heart disease, and other oxidative stress-related diseases, anthocyanins and polyphenols have also been shown to reduce inflammation [81,82]. To that end, PEF treatment was studied in order to examine whether the polyphenol recovery would increase after its use.

Sotelo et al. [83] investigated the impact of low or moderate PEF processing on sweet cherries, focusing on the release of anthocyanins and polyphenols. The electric field strength ranged from 0.3–2.5 kV/cm with a pulse width of 20 μs, pulse frequency of 100 Hz, and pulse number from 385–10,000 with water as solvent. The PEF-treated samples were evaluated immediately and 24 h after treatment. The anthocyanin content, specifically cyanidin glucoside, of the cherry samples was the highest when subjected to high-intensity (2.5 kV/cm) PEF treatment. In addition, it was observed that samples measured 24 h later exhibited a significantly higher content of cyanidin glucoside (~2.3 μg/g wet weight) in comparison to the samples taken immediately after PEF treatment (~1.98 μg/g ww) or untreated samples (~1.95 μg/g ww). This observation indicates that there is a time delay required for the release of anthocyanins following electroporation by PEF. However, an interesting finding was observed in polyphenol extraction, where the increasing PEF intensity resulted in a decrease in recovered polyphenols. For instance, rutin was measured at 7.77 μg/g ww which was much higher than both immediately after PEF measurement (5.04 μg/g ww) and after 24 h of PEF (4.75 μg/g ww).

#### 5.1.2. Grapes

Since 2012, there has been a notable increase in the number of investigations conducted on grape and wine by-products, as well as on grapes and wines themselves. Regarding winemaking, the majority of research studies have focused on investigating the relationship between PEF treatment and the levels of polyphenols present.

El Darra et al. [84] explored the most optimum pretreatment techniques PEF, ultrasound (US), and thermal pretreatment to recover polyphenols from red grapes (Cabernet Franc) during fermentation. The color intensity was increased, the anthocyanins content was raised, and the extraction of phenolic compounds was also increased when thermal pretreatment (50 °C, 125 kJ/kg), US (5–15 min, 121–363 kJ/kg), and PEF (0.8 kV/cm, treatment time of 100 ms, 42 kJ/kg and 5 kV/cm, treatment time of 1 ms, 42–53 kJ/kg) were applied to Cabernet Franc grapes before, during, and after the alcoholic fermentation process. The most effective pretreatments were the moderate (0.8 kV/cm) and high (5 kV/cm) intensity of PEF, which increased anthocyanin extraction yield by 51% and 62%, respectively, from the untreated sample (186 mg/L) while the moderate thermal and US pretreatments increased the yield by 20% and 7%, respectively. Throughout the ethanol fermentation process, wines made from PEF-treated Cabernet Franc grapes maintain their deepest color. It is of high importance that energy expenditure during PEF treatment was the lowest, at 40–50 kJ/kg, while this technique was the most effective one. The phenolic, anthocyanin, and tannin contents of wines could be increased through the US and thermal treatments, and the wine color intensity could be increased in comparison to their untreated counterparts.

A couple of years on, El Darra et al. [85] evaluated the extraction of primary polyphenols and composition (co-pigmentation, non-discolored pigments) of recently fermented model wine from the Cabernet Sauvignon variety using three pretreatments: PEF, enzymes treatment, and thermovinification The application of PEF pretreatment (electric field strength at 5 kV/cm, treatment time of 1 ms, specific energy of 48 kJ/kg) resulted in non-significant variations in the wine polyphenol concentration in the span of 16 days. The untreated sample ranged from initially 130.9 to 305 mg/L, whereas the PEF-treated sample polyphenol content ranged from 364.1 to 359.8 mg/L. Freshly fermented model wines exposed to any of the pretreatments showed increased conversion of anthocyanins to derived pigments. Getting a different profile of newly fermented model wines (color attributes and polyphenol content) without adding additives like enzymes, and especially without heating is the main attraction of PEF pretreatment. When comparing PEF to thermovinification, the former uses less energy (W = 48 kJ/kg) and results in a smaller temperature swing (7 °C compared to 50 °C). However, wines should be tested after months or years of bottling in future studies to further in order for the results to be more valid.

Additional research related to winemaking was carried out by Delsart et al. [86], where Cabernet Sauvignon red grapes were studied. The highest electric field strength 4 kV/cm with a treatment time of 1 ms altered the visual appearance of skin extract to more reddish and led to greater extraction of the anthocyanins from ~480 to ~570 mg/L, while the employment of a lower electric field strength at 0.7 kV/cm and longest treatment duration at 200 ms led to a wine that was richer in tannins by 36% (from 2.5 mg/L) when compared to untreated samples. The parietal tannins and skin cell walls were most affected by the PEF treatment which was both the longest in duration and had the highest energy. Consequently, PEF-treated grapes with low electric field strength for a short time affect primarily anthocyanins, while treatment with a lower intensity but for a much longer time affects mainly tannins.

An interesting research by Delsart et al. [87] examined the evolution of the total polyphenolic index (TPI) at three stages of the vinification cycle (t = 1, 4, 7, and 210 days) in Merlot grapes for both treated and untreated with PEF samples. The results proved that the PEF-treated samples with conditions of an electric field strength of 0.7 kV/cm, and a treatment duration of 40 ms, showed a higher TPC at 210 days. For instance, the PEF-treated sample yielded >950 mg/L, whereas the untreated sample yielded ~870 mg/L of anthocyanins. A similar pattern was observed in tannin concentration, where the PEF-treated samples had a ~18% increase from the untreated samples (~2.7 g/L). However, it was observed that increasing PEF intensity seemed to decrease the tannin concentration, probably due to their diffusion. The pre-fermentative maceration times in winemaking can be shortened as a result of PEF treatment, which accelerates the kinetics of phenolic compound extraction. PEF treatment at 0.7 kV/cm and 40 ms was most preferred based on sensory evaluation. Extraction of intracellular components (total polyphenols, tannins, anthocyanins) was consequently improved with an increase in both electric field strength and pulse duration. However, the enhanced extraction process may compromise the final product’s quality (sensory attributes). The findings also showed that PEF treatment affected aroma composition, which could have significant consequences for the final product aroma and style.

A research study conducted by Maza et al. [88] deals with the dependency of PEF on polyphenol extraction in Grenache grapes. TPI extraction rates were modeled against electric field strength and energy input for each PEF treatment to identify optimal processing parameters for maximizing TPI. All treatments used an optimal energy input of 4 kJ/kg to achieve a TPI of 50, and they varied from high intensity and short time (8 kV/cm and 45 μs pulse duration) to low intensity and long time (1 kV/cm and 2800 μs). The best PEF treatment conditions were 8 kV/cm and 6.7 kJ/kg where a TPI value of 73.15 was ensured while in the control sample, the TPI was 61.15 (about a 19% increase). Maceration time was decreased by 25–37% when these PEF treatments were applied compared to untreated grape samples. The results of this study also suggest that wineries that want to shorten maceration time and still produce high-quality red wines after fermentation and 12 months of aging should apply PEF treatments of 4 kV/cm and 4–5 kJ/kg to the grapes before maceration.

The same research team also conducted a study [89] on Grenache grapes. Garnacha grapes were studied, and the results were compared after 3 and 6 days of maceration and then after 6, 12, and 24 months of bottling. The flow rate was set at 2500 kg/h with a residence time of 0.09 s in the processing area, whereas the PEF treatment amounted to 3.7 square pulses of 100 μs width at an electric field intensity of 4 kV/cm and total specific energy of 6.2 kJ/kg. On day 0, grapes exposed to the PEF treatment and in which the extraction period was the longest (6 days of extraction) showed the highest values of total anthocyanin content (~500 mg/L), TPI (~60 AU at 280 nm), tannin content (~1.5 g/L of epicatechin), as long as color intensity (~15 AU). In a 24-month stability study of flavonoids (anthocyanins, hydroxycinnamic acids, flavonols, flavanols) it was shown that maceration for 6 days was more efficient than maceration for 3 days and then the control sample, measuring the highest flavonoid concentration. The results of this study suggest that the application of PEF treatments has a significant impact on the extraction of various polyphenols and individual polyphenols. Consequently, the wines produced from PEF-treated grapes showed a higher concentration of these compounds compared to wines made from untreated grapes, despite undergoing an equal duration of maceration.

Red wine varieties seem to have piqued the interest of Comuzzo et al. [90]. In Italy, where PEF treatment was evaluated on the modifications of polyphenols in red grapes (cv. *Rondinella*). Regarding the results, color intensity, TPI, anthocyanins, and total tannins recorded the highest values using flow at 250 L/h, the electric field strength at 1.5 kV/cm, pulse length 10 μs (total specific energy 20 kJ/kg) after both two and twelve months of storage. Specifically, these values were found to be 4.3 (38.7% increase), 44.8 (41.3% increase), 78 mg/L (50% increase), and 2.4 g/L (50% increase) after twelve months of bottle storage when compared to the untreated samples, respectively. The same pattern was observed in the HPLC analysis of anthocyanins. This study revealed that PEF-pretreated grapes were able to produce wines of the low-color red cv. *Rondinella* with significantly higher color intensity and stability. After a year of storage, anthocyanin and tannin concentrations were highest in treatments with a specific energy of 10–20 kJ/kg; however, when operating at lower energy levels (2 kJ/kg), adverse consequences were observed. The utilization of PEF technology holds promise in assisting winemakers in the production of consistent varietal wines, without the need to incorporate other (colored) varieties for color correction, thus safeguarding the unique flavor profiles of these wines.

A study [91] conducted in our laboratory proved that applying the PEF pretreatment method is effective only under appropriate electric field intensity conditions. The fact that an increase in electric field strength failed to show a correlation with an improved total polyphenol recovery was of high importance. For instance, upon a range of electric field strength of 1.2–2.0 kV/cm, an intensity of 1.4 kV/cm, and short pulses of 10 μs in a period of 1 ms to fresh grapes (*Vitis vinifera*) led to TPC value of ~110 mg GAE/g dw (49.15% increase) and quercetin-3-rutinoside concentration of 0.083 mg/g dw (85% increase) when compared to the untreated sample. Therefore, this research opens up new horizons regarding the benefits of PEF pretreatment when the optimum conditions are chosen. In the results, it was mentioned that in the same conditions, two more secondary metabolites increased in large percentages after treatment with PEF, kaempferol-3-glucoside and gallic acid which reached 0.153 mg/g dw (66% increase), and 0.124 mg/g dw (63% increase), respectively.

Grape pomace is a by-product that has been extensively studied for the influence of pulsed electric field on the concentration of polyphenols. A corresponding study by Brianceau et al. [92] investigated the extraction kinetics and the level of polyphenols during a hydroalcoholic extraction at different temperatures in fermented grape pomace. In the current investigation, alongside the examination of PEF conditions, the variable of densification pressure was also analyzed. For instance, it was found a PEF pretreatment using electric field strength at 1.2 kV/cm, energy input at 18 kJ/kg, and density at 1.0 g/cm^3^ had a statistically significant (*p* < 0.05) increase in TPC, irrespective of the extraction temperature. It was observed that this density value showed at least 7.5% higher TPC than other values (0.6, 0.8, and 1.3 g/cm^3^). However, a further increase in extraction temperature increased in a higher TPC. For example, gallic acid was increased from 4.53 mg/100 g (20 °C) to 7.40 mg/100 g (50 °C) while, correspondingly, the TPC increase from 60.98 mg/100 g to 113.58 mg/100 g. Total polyphenols extraction from fermented red grape pomace can be improved by densification in conjunction with PEF treatment. Grape pomace that has undergone fermentation can be treated with PEF to enable temperature-selective extraction of total anthocyanins. For these reasons, PEF can be used in place of traditional pre-treatments of raw material (like dehydration and grinding), achieving both goals of lowering production costs and increasing extraction selectivity. This study also sheds light on the possibility of extracting selective phytochemicals from a variety of foods.

The objective of the study by Barba et al. [93] was to assess and compare different solvent-free extraction methods for high-value components in fermented grape pomace. The grape pomace was treated with various physical methods, such as US, PEF, and high voltage electric discharges (HVED), which have the potential to cause cellular damage. These treatments were applied to aqueous suspensions of the pomace. PEF conditions required an electric field strength of 13.3 kV/cm with frequency of 0.5 Hz. The effectiveness of these technologies was evaluated in terms of phenolic compound extraction and, more specifically, anthocyanin recovery while maintaining constant *Z*_p_. According to the results, HVED was found to be the most efficient extraction method. With constant *Z*_p_ set at 0.8, HVED reached ~300 mg GAE/L, almost twice the value of PEF and US. However, in the same *Z*_p_ value, PEF achieved greater anthocyanin recovery than HVED, 63.47 and 40.64 mg/L, respectively. The HVED method demonstrated the highest interest due to its notable impact on polyphenol compound yield. Nevertheless, the selectivity of HVED in terms of anthocyanin recovery was found to be lower compared to that of PEF and US.

To increase the efficiency of the seeds of red grapes (*Vitis vinifera* L.), rich in valuable phenolic compounds, the study by Atanasov et al. [94] discussed the use of low PEF intensity, with electric field strength at 0.86 kV/cm, frequency at 13 Hz, pulse duration 900 μs, pulse interval 75 ms, and treatment time 810 ms. By applying PEF to the red grape samples, similar polyphenol yields could be achieved with lower concentrations of ethanol. For instance, comparable results (~24 mg GAE/g) were measured when an untreated sample was extracted for 120 min with 75% ethanol and a PEF-treated sample was extracted with 20% ethanol. Therefore, it is possible to enhance the release of thermally unstable bioactive compounds under mild processing conditions by optimizing electric field strength in combination with an appropriate solvent system.

Delso et al. [95] assessed the viability of PEF technology as a potential alternative approach for the processing of red grape juice, so they studied the enhancement of juice derived from grapes. The sample was treated with PEF (electric field strength at 5 kV/cm, specific energy at 63.4 kJ/kg, and pulse width 40 μs) and had a TPC 1.5 times higher than that of untreated grapes, 1434.30 mg GAE/L and 916.10 mg GAE/L, respectively. The same pattern was observed in every antioxidant assay (TPI, CI, antioxidant capacity, and 2,2-diphenyl-1-picrylhydrazyl, DPPH^•^). The study also contained a decontamination process, in which the PEF conditions included electric field strength at 17.4 kV/cm, specific energy at 173.6 kJ/kg, and pulse width of 10 μs. With PEF treatment, the current microbiota was eliminated to an undetectable level (30 CFU/mL) of yeasts, molds, and vegetative mesophilic bacteria. Furthermore, even at abusive refrigeration storage temperatures (10 °C), PEF-treated juices were microbiologically stable for up to 45 days. The PEF decontamination treatment and storage time/temperature did not affect the juice’s quality or sensory characteristics, which were similar to those of fresh juice. This study highlights the promising future of PEF as a sustainable, enzymatic and heat-free alternative for the production of polyphenol-enriched and microbially stabilized red grape juice in the juice industry.

In a study by Ricci et al. [96] the impact of PEF treatment was investigated on the extractability of anthocyanins and polyphenols in Sangiovese red grapes. The grapes underwent a pre-fermentative PEF treatment on a pilot scale, with electric field strengths in the range of 0.9–3 kV/cm, generated by the application of short, high-voltage pulses, and specific energies from 10.4–32.5 kJ/kg. The results showed that a PEF treatment with dynamic maceration for 2 h, and static maceration for 12 h led to the highest polyphenol recovery at 439 mg GAE/L, achieving ~55% increase from the untreated sample.

Ziagova et al. [97] focused on the recovery of polyphenols from grape leaves and marc from cv. *Xinomavro*, which was examined for its antioxidant activity and TPC. PEF was combined with US-assisted extraction. The most effective results were achieved when a solid-to-liquid ratio of 1:20 of plant material and water as solvent were used, 5 min of PEF at an electric field strength of 0.5–2 kV/cm, and 30 min of US. Comparing grape leaves and marc, it was found that grape leaves were richer in polyphenols (97 and 31 mg GAE/g dw, respectively). The same pattern was observed in the antioxidant capacity levels were 88% was observed for grape leaves and 31% for grape marc.

The study conducted by Ntourtoglou et al. [98] examines the impact of PEF treatment on stem grapes, specifically focusing on the increase of polyphenols and volatile compounds. A PEF process with a relatively low electric field strength of 1 kV/cm, for a brief duration of 30 min on the grape stems. When PEF was solely used as an extraction technique, the extracted TPC was measured at ~0.05 AU and showed a statistically non-significant increase (only 4%) regardless of the solvent (50% *v*/*v* aqueous methanol or water). With the implementation of US extraction, the samples with 50% *v*/*v* methanol reached a 17% increase, whereas samples with water as a solvent had a 35% increase, revealing the importance of extraction solvent. Regarding volatile compounds, the control sample showed an average concentration of 0.73 mg/Kg. However, when PEF was applied before US extraction, the observed increase in concentration was as significant as 234%. The fact that two more volatile compounds (Benzene, 1-methoxy-4-methyl, and 1,14-Tetradecanediol) were extracted is of high importance. In conclusion, the utilization of PEF as a preliminary treatment method for extracting different volatile and polyphenolic compounds shows significant potential for improving the efficiency of the extraction process. On top of that, through the examination of additional factors related to the extraction process, such as the selection of extraction solvents, duration, and temperature, it may be possible to further optimize the extraction yield.

The most recent study was conducted by Carpentieri et al. [99]. Total polyphenols, tannins, anthocyanins, and flavonoids were extracted from red grape pomace using PEF, and response surface methodology was used to analyze the efficacy of this process in improving the extraction of these crucial intracellular components. Results showed that grape pomace tissue permeability was significantly increased after PEF was applied under optimal processing conditions (electric field strength of 4.6 kV/cm and energy input of 20 kJ/kg). In comparison to the control (PEF-untreated sample), this increased extraction rates of TPC by 15%, TFC by 60%, total anthocyanin content (TAC) by 23%, and tannins content (TC) by 42%. Additionally, it was observed that PEF treatment did not result in the degradation of these compounds.

#### 5.1.3. Apples

An apple is the fruit of the apple tree of the Rosaceae family. It is by far one of the world’s most widespread and widely grown fruits since it represents 50% of deciduous fruit trees, with a global yearly output of about 60 million tons. Apples are rich in polyphenols and mainly flavanols (catechins and proanthocyanidins), which are also considered the main category of polyphenols in apples (71−90%), followed by hydroxycinnamic compounds (4−18%), flavonols (1−11%), dihydrochalcones (2−6%) and anthocyanins (1−3%) [100].

The objective of the study from Wiktor et al. [101] was to investigate the impact of PEF treatment on the concentration of particular bioactive compounds from apple tissue. The range of specific energy input varied between 0–80 kJ/kg. Apple tissues were subjected to PEF treatment at different electric field strengths of 0, 1.85, 3, and 5.0 kV/cm, and at varying pulse numbers of 0, 10, 50, and 100. The results showed that at 3 kV/cm and 100 pulses, a slight increase of ~10% in TPC of apple tissues was feasible compared to the untreated sample (426.69 mg chlorogenic acid/100 g dm). The findings of the study suggest that PEF has the potential to improve the efficiency of extracting bioactive compounds from plant tissue.

In a study by Dziadek et al. [102], a PEF treatment with a number of cycles ranging from 4, 6, and 8 (200, 300, and 400 pulses, correspondingly) was used in apple juice. The concentration of polyphenols and antioxidant activity were all measured immediately after the PEF process after 24, 48, and 72 h of refrigeration. The electric field strength was set at a high value of 30 kV/cm. The untreated sample stored for 0 h yielded 337.51 mg/100 mL and it was observed that the application of PEF treatment, irrespective of the pulse quantity, did not yield a statistically significant impact on the overall polyphenol content found in apple juice. Antioxidant activity was measured at 17.4 μmol Trolox/mL and was found to be reduced both immediately after processing and after 24 h of storage, both of which were affected by PEF treatment and the number of pulses. However, increasing PEF pulses were observed to increase polyphenol concentration after 24, 48, and 72 h of refrigeration, from ~233 to 300.90, 280.88, and 295.66 mg/100 mL.

PEF treatment was also studied in apple by-products. A study by Pollini et al. [103] in 2021 examined the effect of various non-conventional extraction methods, such as PEF, ultrasound-assisted extraction (UAE), supercritical fluid extraction (SFE), microwave-assisted extraction (MAE), and pressurized liquid extraction (PLE) on the polyphenol content in apple pomace. Only two PEF pilot treatment experiments were performed to benchmark a short-term, medium-intensity treatment (3 kV/cm and 9 pulses with 450 J energy/pulse) against a longer, low-intensity treatment (2 kV/cm and 100 pulses with 200 J energy/pulse). Hydroethanolic solutions from 30–70% *v*/*v* were used as extraction solvents. Regardless of the PEF treatment used, the values of polyphenols ranged from 181.4–223.5 μg GAE/g of fresh apple pomace. However, better values were obtained using 30% *v*/*v* aqueous EtOH as a solvent in both PEF treatments. Nevertheless, it was observed that the apple pomace samples treated with PEF had the lowest results in terms of polyphenol values compared to other treatments.

Recent research on the apple fruit was carried out by Matys et al. [104] who combined the MAE with PEF treatment to dry apples. The electric field strength was set at 1 kV/cm, pulse frequency at 20 Hz, and pulse width at 7 μs. The total amount of rectangular pulses had supplied energy of 1, 3.5, and 6 kJ/kg, whereas microwave power ranged from 100–300 W. The results showed that in high energy input (6 kJ/kg), the cell disintegration index was measured at 0.36. However, when specific energy was set at 3437 kJ/kg and 300 W of microwave power through response surface methodology, the results showed that the desirability was found to be 0.624, whereas total polyphenols were predicted to be 1257 mg GAE/100 g dm.

#### 5.1.4. Pomegranate

*Punica granatum*, the pomegranate’s scientific name, stems from the Latin for seeded apple, *Pomum granatus* [105]. The pomegranate is a plant of the genus Punica, family Punicaceae, and a source of high content of phenolic components with highly bioactive characteristics [106].

PEF pretreatment was applied to the pomegranate peel in a study by Rajha et al. [107], who used a variety of extraction techniques, such as infrared (IR), US, PEF, and high voltage electric discharge (HVED). For all extractions, the temperature was held at 50 °C. The electric field strength was maintained at 10 kV/cm, with the temperature rise during PEF treatment being kept below 5 °C and the total input energy ranging from 90–100 kJ/kg. The polyphenol extractable fraction by PEF showed a mean value of 39.2 mg GAE/g dm after 7 min of extraction. When compared to the HVED treatment, this value was found to be 15.22% lower. In comparison to other treatments, however, it was observed to be 168.97% more effective than the US, 387.5% more effective than IR, and 680% more effective than a water bath treatment. Comparing this method to Ziagova et al. [97] who also used a combination of PEF and US pretreatments, the latter authors measured 208 mg GAE/g dw of pomegranate peels. This research shows that both the HVED and PEF treatments are highly efficient at removing polyphenols from pomegranate peels. PEF is less efficient at polyphenol recovery than HVED. In contrast to HVED, the treatment process in PEF is more selective and causes less damage.

#### 5.1.5. Citrus Fruits

Citrus fruits such as oranges (*Citrus sinensis*), lemons (*Citrus limon*), and pomelos (*Citrus maxima*) are the most valuable fruit crops worldwide. The vibrant color, delicious flavors, and refreshing aromas of citrus fruits have made them a global favorite. Antioxidant bioactive compounds eliminate free radicals, halt lipid peroxidation reactions, and protect against other forms of oxidative damage, all of which are essential to the proper functioning of a cell. Moreover, their antioxidant activity may protect against a wide range of chronic diseases like cancer, diabetes, and heart disease [108].

The processing of citrus fruits is of industrial significance because they are rich in bioactive compounds (such as vitamins, antioxidants, carotenoids, and polyphenols). Juice extraction from various citrus fruits could benefit from PEF being applied to whole fruits. In addition, PEF-treated citrus peels at high electric field strength could significantly increase the concentration of polyphenols in the extracted juice. To that end, a corresponding study was conducted by El Kantar et al. [109], who intended to improve polyphenol extraction and improve the juice. The experimental procedure involved subjecting unharmed fruits and a pile of peels to PEF treatment, with electric field strengths of 3 kV/cm and 10 kV/cm, respectively. The samples were then subjected to 1 h of extraction with 50% *v*/*v* aqueous ethanol solution. The implementation of PEF increased extracted polyphenols in citrus fruit juice. Orange, lemon, and pomelo TPC were measured at ~70 mg/100 mL (~49% increase), ~60 mg/100 mL (~50% increase), and ~80 mg/100 mL (~60% increase) when compared to the untreated samples. Furthermore, PEF treatment affected major polyphenols concentration from orange (hesperidin) and pomelo (naringin) flavedo, which were increased from 4.852 to 5.073 mg/g of dried mass and from 7.354 to 10.366 mg/g of dm, respectively. An interesting decrease of eriocitrin in lemon flavedo was observed from 3.064 to 1.440 mg/g dm after PEF treatment.

Since orange peels (albedo and flavedo) contain ascorbic acid, carotenoids, and polyphenols, Athanasiadis et al. [110] set out to investigate and optimize the factors affecting the extraction process. They used US or PEF as a pretreatment step prior to extracting the bioactive compounds using an ethanol and water mixture. In this experiment, the authors used an electric field strength of 1 kV/cm, pulse duration of 10 μs, and treatment period of 1 ms (frequency: 1 kHz). The extraction time (15–180 min) and temperature (20–80 °C) were optimized using a response surface methodology. Hesperidin (16.26 mg/g dw) and TPC (34.71 mg GAE/g dw) were improved by PEF treatment. The study may have flaws, such as its narrow focus on a single orange variety. For real-world, large-scale applications, however, the proposed pretreatment methods involving PEF hold much promise. These applications span numerous sectors, including the food and beverage industry, the cosmetics and pharmaceutical industries, and the movement to replace synthetic pigments with natural ones.

Luengo et al. [111] conducted a study to investigate the PEF treatment on the extraction of polyphenols from orange peel during the pressing process. The highest value of *Z*_p_ was obtained when various electric field strengths were tested, with a treatment time of 60 s (consisting of 20 pulses, each lasting 3 s). Treatments with PEF consisted of 5–50 pulses of 3 s each (15–150 s) at electric field strengths of 1–7 kV/cm. The treatments varied in the amount of energy they provided, from 0.06–3.77 kJ/kg. Distilled water was used as the extraction solvent. The frequency of the pulses was set to 1 Hz. Following exposure to PEF with an electric field strength of 5 kV/cm, the concentrations of naringin and hesperidin exhibited a significant increase, rising from 1 to 3.1 mg/100 g fw and 1.3 to 4.6 mg/100 g fw, respectively. Furthermore, the extraction yield of polyphenols was determined to be 34.80 mg/100 g fw. The application of PEF to orange peel resulted in a significant increase in the yield of polyphenol extract. Specifically, at electric field strengths of 1, 3, 5, and 7 kV/cm, the yield of polyphenol extract increased by 20, 129, 153, and 159% respectively. The application of PEF treatments at intensities of 1, 3, 5, and 7 kV/cm resulted in a significant enhancement of the antioxidant activity in the extract. Specifically, the antioxidant activity increased by 51, 94, 148, and 192% compared to the untreated sample, respectively. The utilization of PEF technology presents numerous advantages in comparison to alternative techniques employed for enhancing the extraction of polyphenols from orange peels via pressing. These advantages include the elimination of the requirement to dehydrate the sample and the utilization of water as the solvent. In addition, this method presents an environmentally friendly and cost-effective alternative to conventional extraction techniques, such as initial product dehydration and the utilization of substantial quantities of organic solvents.

The Interest of the researchers was also directed towards the peel of lemons. Total polyphenols were extracted from lemon peel residues by pressing, and Peiró et al. [112] studied the effect of different PEF intensities (3–9 kV/cm and 0–300 s treatment time pulses). Since no statistically significant differences were found between 7 and 9 kV/cm, *Z*_p_ concluded that 30 pulses of 3 μs (total 90 μs) and an electric field strength of 7 kV/cm are optimal for increasing permeability. Compared to the untreated samples, PEF did not affect the polyphenol extraction yield regardless of the size of the lemon residue (1, 2, or 3 cm). However, 3-cm lemon peels were chosen as the optimal size, and PEF treatment significantly increased TPC, as an average of 160 mg GAE/100 g of dw was up to a 150% increase from the control sample. Pressure and electric field strength were found to significantly increase the concentrations of hesperidin and eriocitrin, the two most abundant polyphenols in lemon residues. Applying the pulsed electric field with 30 pulses of 30 μs and electric field intensity of 7 kV/cm, the extraction efficiency of polyphenols showed a 300% increase, as well as hesperidin, reached 84 mg/100 g fw and the amount of eriocitrin was 176 mg/100 g fw.

The purpose of the study by Chatzimitakos et al. [113] was to determine the most efficient conditions for extracting bioactive compounds from lemon peel waste. Time, temperature, and solvent composition were among the variables tested alongside a number of different extraction methods like stirring, US, and PEF. The electric field strength was set at 1.0 kV/cm with a pulse period of 1 ms, and 10 μs pulse length. The results revealed a negative impact of PEF in TPC yield, rendering sole stirring as the optimum condition for extraction with 50% *v*/*v* aqueous ethanol for 120 min at 50 °C. It was also observed that PEF with US had a deleterious effect on polyphenol extraction compared to conventional extraction. However, PEF treatment prior to stirring with 100% ethanol for 60 min at 80 °C resulted in the highest TFC yield at 7 mg RtE/g. 

#### 5.1.6. Quince

Leaves and peels are quince by-products. Recovery of bioactive compounds from quince peels was investigated by Athanasiadis et al. [114]. Extraction parameters, such as solvent, temperature, and time, as well as extraction techniques such as PEF and US, were initially investigated for their effects. Then, these parameters were optimized using response surface methodology to extract bioactive compounds more effectively. The PEF used an electric field strength of 1 kV/cm, had a pulse duration of 10 μs, a pulse period of 1 ms, and a frequency of 1 kHz. Using a frequency of 3 kHz and a bath temperature of 30 °C, US was applied for 20 min. The best extraction conditions were achieved by stirring at 65 °C for 120 min. Using techniques like principal component analysis and partial least squares analysis, authors were able to establish that quince peels contain a large number of bioactive compounds. TPC (43.99 mg GAE/g dw), TFC (3.86 mg RtE/g dw), and chlorogenic acid (2.12 mg/g dw) were some of these. Using FRAP and DPPH^•^ assays, they found that the quince peels had 627.73 and 699.61 mol AAE/g dw, respectively, of antioxidant activity.

#### 5.1.7. Berry Fruits

Lončarić et al. [115] aimed to determine the specific conditions of PEF that would result in a higher concentration of polyphenols and flavonoids in blueberry pomace. Applying PEF-assisted extraction, where ethanol was used as a solvent, with 100 pulses and an intensity of 20 kV/cm, which equals a specific energy input of 41.03 kJ/kg, a vast amount of TPC was extracted (10.52 mg GAE/g dw). Additionally, with the previous PEF conditions, phenolic acid and flavonol concentrations displayed their highest values (625.47 μg/g dw and 157.54 μg/g dw, correspondingly). On the contrary, when methanol was used as a solvent, anthocyanin and flavanol best amount of field noted (1757.32 μg/g dw and 297.86 μg/g dw, respectively). Upon comparison of other green extraction methods, it was evident that the total phenolic content (TPC) of PEF-treated extracts exhibited higher values in comparison to those obtained through HVED (~5 mg GAE/g dw) and US methods (~6 mg GAE/g dw).

The nutritional properties of red raspberry (*Rubus strigosus* var. *Meeker*) and blueberry (*Vaccinium corymbosum* var. *Bluejay*) purees after US and PEF exposure were investigated by Medina-Meza et al. [116]. When PEF-assisted extraction was applied with a high electric field strength of 25 kV/cm, and flow rate at 300 mL/min along with US treatment, no deleterious effects were observed. In relation to red raspberry, solely PEF treatment resulted in non-significant differences (*p* < 0.05) in TFC, whereas when combined with US led to an increase of ~20% from an initial ~150 μg/mL of quercetin. In PEF-increased anthocyanin concentration from ~125 to ~145 mg/L, however, a deleterious effect and decrease of at least 66% was observed with US treatment, which requires further investigation. Regarding TPC content, PEF increased the recovery from ~430 to ~470 μg/mL. In blueberry samples, PEF treatment had roughly any impact on the extraction of flavonoids (~310 μg/mL) but increased the anthocyanins recovery from ~650 to ~750 mg/L. However, another deleterious effect was observed in TPC, where a decrease of ~6% was measured from an initial ~520 μg/mL. 

Ozkan et al. [117] investigated non-thermal processing methods of high-pressure processing (HPP) and PEF to preserve polyphenols in cranberrybush (*Viburnum opulus*) puree. This investigation required PEF conditions, including 20 μs pulse width, an electric field strength of 3 kV/cm, pulse frequency of 2 Hz, and specific energy input of 15 kJ/kg. The application of PEF or HPP resulted in enhanced preservation of bioactive compounds, as evidenced by a significant increase in TPC, ranging from 4.1–14% (from ~400 mg GAE/100 g fw). Additionally, both treatments demonstrated improved antioxidant activity in CUPRAC (~7% increase from 1500 mg TE/100 g fw). The results of this study suggested that the PEF process was successful in non-thermal treatments for the extraction of polyphenols from cranberry bushes.

The objective of the study from Gagneten et al. [118] was to enhance the extraction of polyphenols from blackcurrants through the utilization of PEF treatment. The study aimed to analyze the effects of electroporation on biological samples at two different initial temperatures, specifically 10 and 22 °C. A small quantity of the natural fruit juice is used to hydrate the sample. The optimal PEF conditions for achieving the greatest benefits were found to be an electric field strength of 1318 V/cm and a total of 315 pulses. The study examined the influence of electric field strength and treatment duration on the TPC and AA. Under optimum conditions, notable improvements were observed, including a 19% increase in TPC (from 3.18 mg GAE/g extract), a 45% increase in antioxidant activity (AA) (from 1.12 mg GAE/g extract), and a 6% increase in total monomeric anthocyanins (from 1.30 mg cyanidin-3-glucoside/g extract). The application of PEF treatment to blackcurrant juices resulted in a higher efficiency in extracting bioactive compounds, making them a promising choice for incorporation as functional food ingredients.

In light of the demonstrated beneficial health effects of polyphenols, Stübler et al. [119] examined the impact of various processing methods (thermal, PEF, HPP) as well as the incorporation of a vegetable juice (kale) with a relatively high protein content on the stability and bioaccessibility of polyphenols in strawberry puree. PEF conditions required an electric field strength of 11.9 kV/cm with 20 μs pulse width and energy input of 120 kJ/kg. As for the results, it was found that anthocyanins showed an increase both in the strawberry–kale mix and in the puree. Specifically, anthocyanins content increased from almost 32 (control) to 35 mg pelargonidin-3-glucoside/L (PEF-treated) in the mix and 40 (control) to 45 mg pelargonidin-3-glucoside/L (PEF-treated) in the strawberry puree. The industrial significance of the current trend towards healthier eating habits and fast-paced lifestyles is evident in the increased demand for convenient and nutritious food options centered around fruits and vegetables. Insufficient data exists regarding the effects of alternative processing methods on multi-component juice systems, wherein interactions between various components may transpire. Nevertheless, these methods are regarded as a promising means of producing nutritionally rich products. Strawberries, being the berry with the highest consumption rate, offer a substantial quantity of polyphenols and serve as an exemplary system abundant in polyphenolic compounds.

The effects of PEF on tomato juice and fruit were studied. Firstly, the purpose of the study by Vallverdú-Queralt et al. [120] was to examine the impact of using moderate (MIPEF) and high-intensity PEF (HIPEF) treatments in succession on the polyphenol profile of tomato juices. A variety of intensities in the electric field were used to test the effects of different nutrients on tomato fruits. MIPEF conditions required 1 kV/cm of electric field strength and 16 pulses of 4 μs at a frequency of 0.1 Hz, whereas HIPEF included 35 kV/cm and 4 μs pulses with a frequency of 100 Hz. Tomato juices made from untreated tomatoes had an initial TPC of ~148 μg/g fw, while juices from MIPEF- and HIPEF-treated tomatoes had an initial TPC of ~180 and ~155 μg/g fw. The polyphenol amount that was gained was enhanced by 44% with the addition of 30 pulses at 1.2 kV/cm. Therefore, it may be possible to propose the use of MIPEFs and HIPEFs together as a method for increasing the phenolic content of tomato juices. The same research team [121] studied the impact of PEF on TPC and the antioxidant capacity of tomato fruit. Electric field strength from 0.4–2.0 kV/cm and the number of pulses from 5–30 were investigated. At 1.2 kV/cm and 30 pulses, TPC achieved 144.61% relative TPC (44% increase). With the same electric field strength (1.2 kV/cm) but with fewer pulses (18), a higher % relative hydrophilic antioxidant capacity was achieved (131.75%).

#### 5.1.8. Red Prickly Pear

Surano et al. [122] studied the use of PEF in a multiple-needle chamber to enhance the extraction of bioactive compounds from raw *Opuntia ficus-indica* (red prickly pear) fruits. The high concentration of natural pigments and bioactive compounds in prickly pear fruit contributes to its antioxidant capacity and health-promoting properties. In order to accomplish polyphenol recovery with a non-thermal technique, a novel electroporation chamber was designed and implemented, to identify the optimal pulse parameters that would maximize both juice yield and the extraction of bioactive compounds. To achieve optimum PEF conditions, it was necessary to apply pulses with an electric field strength of 1200 V/cm and a frequency of 10 Hz. A mean value of specific energy input at 11.44 kJ/kg was utilized, resulting in a temperature rise of less than 10 °C. PEF-treated samples resulted in a significant increase in juice yield by a factor of 3.3 (from 16.69%) and betalain extraction by a factor of 1.48 (from 19.5 mg/100 g), as compared to the samples that did not undergo PEF treatment. The extracted juice exhibited a notable enhancement in its antioxidant capacity, with an increase of approximately 1.4–1.5 times, measured with three different methods (DPPH^•^, ABTS, FRAP). Similarly, the TPC of the juice experienced a proportional increase of 1.4 times (from 9.49 mg GAE/100 g). The chamber could accommodate sizable samples without necessitating the inclusion of a conductive medium or the removal of fruit peels. This simplifies the procedure and leads to cost reduction, thereby positioning it as a viable option for industrial applications. The application of PEF treatment on prickly pear fruits results in the production of juice that exhibits enhanced nutritional content and a greater abundance of naturally occurring pigments compared to juice derived from untreated fruits. Table 1 provides a concise summary of the above research on fresh fruits and by-products.

### 5.2. Vegetables

#### 5.2.1. Potato

The fourth most important food grown and consumed in the world, potatoes are a staple that can be found in almost every region of the country [123]. They are rich in carbohydrates and have a small amount of fat. They are also known to contain various nutrients such as vitamins, proteins, and fiber. Bioactive compounds such as anthocyanins and polyphenols are commonly found in the skin and flesh of potatoes [124].

To improve the extraction of polyphenols with significant antioxidant properties from potato peels, Frontuto et al. [125] studied the optimal PEF-assisted extraction conditions. To verify this, they applied electric field strength from 1–5 kV/cm and total specific energy inputs from 1–10 kJ/kg. Total polyphenol yield from PEF pretreated sample extracts was 1295 mg GAE/kg fw (+10% from the control) when using 5 kV/cm fields strength and 10 kJ/kg specific energy output, 52% ethanol as a solvent, 230 min of extraction time, and 50 °C for the subsequent solid-liquid extraction. The HPLC-DAD analysis revealed that the predominant polyphenolic compounds detected were chlorogenic, caffeic, syringic, protocatechuic, and *p*-coumaric acids. There was no indication of substantial degradation of these individual polyphenols as a result of the application of PEF.

#### 5.2.2. Asparagus

Beneficial bioactive compounds have been found in *Asparagus officinalis* root. The primary goal of the research conducted by Symes et al. [126] was to improve the efficiency with which polyphenol and flavonoid extraction was accomplished from the roots of green asparagus by combining PEF and ionic liquids. Antioxidant activity (oxygen radical absorbance capacity, ORAC), FRAP and DPPH^•^), TPC, and TFC were all measured in this study. When compared to the standard solvent extraction method, the extraction yield was higher when PEF was used under the optimum conditions of electric field strength at 1.6 kV/cm, pulse width of 20 μs, and frequency of 200 Hz. All assays, except for ORAC, showed improvements in PEF-treated samples compared to untreated samples. Extraction yield was increased by 23%, TPC by 5%, TFC by 6%, and FRAP by 4%. Ionic liquids, on the other hand, were discovered to be more efficient than PEF treatment. For instance, ionic liquids were found to have a TFC of 122 mg RE/g. This value was ~80 times greater than the TFC achieved by PEF treatment. While ionic liquids performed better than PEF in asparagus root samples, more research is needed to determine their safety for use in the food industry.

#### 5.2.3. Mushroom

Mushrooms are frequently consumed as a staple in vegetarian diets. They are recognized for their wide variety of health advantages, including their anti-carcinogenic and anti-infectious attributes. Furthermore, the abundant presence of polyphenols in these substances renders them highly versatile for utilization as pharmaceutical agents or dietary supplements [127,128]. The only type of mushroom that was applied to the PEF system with the purpose of greater extraction of polyphenols was *Agaricus bisporus*.

The utilization of chemical or thermal methods for the extraction of valuable compounds is widely employed across various disciplines. The utilization of PEF during the extraction process reduces the likelihood of nutrient damage in the extracted product. To that end, the study by Xue et al. [129] aimed to investigate the impact of continuous PEF treatment on the extraction process of a white button mushroom suspension with a concentration of 9% *w*/*w*. PEF with intensities ranging from 12.4–38.4 kV/cm were applied using bipolar square pulses lasting 2 μs. The mushroom suspension was exposed to electric pulses with a field intensity of 38.4 kV/cm for a duration of 272 μs at 85 °C. Based on estimations, it was determined that the optimal extraction yields would be 98% (7.9 mg/g mushroom) of polysaccharide, 51% (1.6 mg GAE/g mushroom) of total polyphenols, and 49% (2.7 mg/g mushroom) of proteins. However, traditional mushroom extraction methods yielded only 56% (6.4 mg/g of mushroom) polysaccharide, 25% of total polyphenols (1.3 mg GAE/g of mushroom), and 45% (2.6 mg/g of mushroom) proteins after processing the 9% *w*/*w* mushroom suspension at 95 °C for 1 h. For all of these substances, the yield from conventional extraction carried out at the same temperature and for a similar amount of time was negligible. This indicates that a synergistic effect of electric pulses and temperature on the extraction performance is responsible for the improvement in extraction performance observed with PEF and that this improvement cannot be attributed solely to ohmic heat generated during PEF treatment.

#### 5.2.4. Olives

Olives of several cultivars are key in Mediterranean dishes and an essential agricultural crop for European countries such as Greece, Spain, and Italy. Olives are the fruit of the olive tree with the scientific name *Olea europaea*, which means “European olive”. They are cultivated throughout the Mediterranean basin as well as in South America, South Africa, India, China, Australia, New Zealand, Mexico, and the United States. Antioxidant compounds, such as polyphenols and flavonoids, are characteristic in olives, especially oleuropein. The content of extra virgin olive oil in polyphenols is 500 mg/L [130]. It has been claimed that the health benefits of olives and olive oil can protect the human organism from a variety of illnesses [131]. For this reason, the effect of PEF pretreatment on the increase of their polyphenol content was evaluated for all parts of the olive plant as well as for extra virgin olive oil. For instance, in the previously mentioned study by Ziagova et al. [97], PEF-treated leaves and unripe fruit yielded TPC of 105 and 12 mg GAE/g dw, respectively.

Andreou et al. [132] improved the recovery of high value-added compounds from olive pomace as a result of the combined use of PEF. Pretreatments with PEF (1.0–6.5 kV/cm, 0.9–51.1 kJ/kg, and 15 μs pulse width) were applied to olive pomace (cv *Tsounati*). Solid-liquid extraction of intracellular compounds with 50% *v*/*v* aqueous ethanol solution for 1 h at 25 °C was then used for analysis. At electric field strengths over 3 kV/cm, the polyphenol concentration increased significantly, reaching as high as 91.6% from the untreated sample (~1500 mg GAE/L). This method may be useful in conjunction with traditional solvent extraction. With PEF pretreatment, olive pomace can be valorized by increasing the yields of intracellular compounds with high antioxidant properties.

The same research team [133] investigated the application of non-thermal processing techniques to maximize the production and quality of virgin olive oil. Different PEF conditions (electric field strength of 0.5–2.0 kV/cm, 0–2500 pulses, energy input of 0.5–57.5 kJ/kg) were applied to olive paste. Quality, bioactive compounds, oxidative stability, and sensory evaluation of olive oils generated under optimum conditions (electric field strength of 1.5 kV/cm, 100 pulses) were assessed using response surface methodology. The olive oil yield from the PEF-pretreated sample was ~3% higher (25.4%), had ~57% more α-tocopherol (66.9 mg/kg oil), and had ~7% more polyphenols (822.3 mg GAE/kg oil) than the yielded oil from the untreated control sample. Consequently, PEF seeks to increase the production of a sustainable and cost-effective product in the olive oil industry.

Extracting polyphenols from olive leaves using the PEF method was evaluated by Pappas et al. [134]. Water, ethanol, and various mixtures thereof were used across a gradient of 25% in this study. The optimal conditions for PEF extraction took 30 min and required an electric field strength of 1 kV/cm. In addition, they explored a range of pulse durations, from 10–100 ns. Results obtained from PEF-treated extracts were compared to those obtained from untreated extracts. With a pulse duration of 10 μs and a 25% *v*/*v* ethanol aqueous solution, PEF was found to have the greatest effect. Significant increases of 31.85% in total polyphenols and 265.67% in specific metabolites were observed when comparing pre- and post-harvest samples. Differential scanning calorimetry revealed that 569 °C was the highest temperature at which oxidation occurred. The higher the oxidation temperature, the more resistant to oxidation the sample. This remarkable temperature was achieved by subjecting the samples to pulses with a duration of 100 μs and a period of 1000 μs. When the above PEF parameters were applied, the main metabolite luteolin-7-*O*-glucoside showed a significant increase of 71.87%, amounting to a total of 0.82 mg/g dw. Under a 100 μs pulse, however, oleuropein alone showed the highest extraction yield.

The same research team conducted an investigation [135] into the efficacy of PEF in concentrating polyphenol extracts, thereby evaluating the potential worth of olive leaves. The researchers thoroughly investigated the optimal methods for enhancing the PEF process in order to improve the extraction of olive leaf compounds. A comprehensive investigation was conducted to examine various parameters of the extraction chamber, including its geometric configuration, electric field intensity, pulse duration, pulse period (and frequency), and extraction duration. The authors employed experimental methods to ensure the optimal duration of PEF-assisted solid-liquid extraction of olive leaves. The implementation of PEF resulted in a significant increase of 38% in the extractability of total polyphenols compared to the untreated control sample. The TPC reached a value of 25.35 mg GAE/g dw. Furthermore, it is noteworthy to mention the remarkable 117% increase observed in the concentration levels of certain specific metabolites. The optimum conditions required a 15-min extraction with 25% *v*/*v* ethanol conducted in a rectangular extraction chamber, an electric field strength of 0.85 kV/cm, a pulse period of 100 μs, and a pulse duration of 2 μs. Regarding oxidative stability, the samples subjected to a pulse duration of 10 μs, pulse period of 1000 μs, electric field of 0.85 kV/cm, and extraction time of 30 min exhibited the most pronounced oxidation peak at 488 °C. This value was found to be 16% higher than the control sample when assessed using differential scanning calorimetry. The findings of this study indicate that the use of PEF treatment has a notable impact on enhancing extraction efficiency and improving the physicochemical properties. Table 2 provides a concise summary of the above research conducted on fresh vegetables and their by-products.

### 5.3. Various Plants, Herbs, Nuts and Seaweeds

A variety of aromatic plants, herbs, nuts, plant parts, and seaweeds were utilized in the process of PEF-assisted extraction. The analysis of all the samples revealed a notable increase in their polyphenol levels, with certain samples exhibiting a more pronounced increase compared to others.

#### 5.3.1. Borage

The objective of the study by Segovia et al. [136] was to evaluate the efficacy of PEF in enhancing the aqueous extraction of polyphenols and antioxidant compounds from borage (*Borago officinalis*) leaves. The range of the applied electric field strength ranged from 0–5 kV/cm. Extractions were exposed to different temperatures (10, 25, and 30 °C) for varying durations (10–60 min), whereas water was used as a solvent. The application of PEF treatments resulted in a significant increase in the TPC and ORAC values of the extracts. Specifically, TPC values were enhanced by a factor ranging from 1.3–6.6 (from 0.3 mg GAE/g fw), while the ORAC values were enhanced by a factor ranging from 2.0–13.7 (from ~10 mg TE/g fw). This procedure enhances the efficiency of the extraction process while concurrently increasing the antioxidant potency of the extracts. Furthermore, it reduces the duration of the extraction process. The correlation between the rise in pulse intensity and the quantity of extracted polyphenols indicates that this technology holds promise for effectively managing this byproduct within the food industry.

#### 5.3.2. Flaxseed

Boussetta et al. [137] investigated the polyphenol extraction from flaxseed hulls using PEF as part of an effort to identify potential uses for oil-seed byproducts. The study examined the influence of various factors, including PEF treatment time, electric field strength, composition of solvent, and duration of rehydration, on the extraction of polyphenols. The extraction efficiency of polyphenols (~314 mg GAE/100 g dm) was achieved by 50% *v*/*v* ethanol, reaching an increase of 42% compared to extraction with 20% *v*/*v* ethanol. However, this ethanol level (20% *v*/*v*) was used as optimal because the authors stated that industrial PEF applications should not exceed this level. After 40 min of rehydration at 150 rpm, the study found that the optimal conditions for a PEF treatment were 20 kV/cm for 10 ms with an energy output of 300 kJ/kg. While 270 mg GAE/100 g of dm was recovered using 0.3 M citric acid, 1000 mg GAE/100 g of dm was recovered using 0.3 M sodium hydroxide. The sodium hydroxide-free sample yielded about 200 mg GAE/100 g dm. Consequently, the use of PEF in conjunction with alkaline hydrolysis gave encouraging results in polyphenol recovery.

#### 5.3.3. Rapeseed

Yu et al. [138] examined the utilization of PEF for the extraction of polyphenols from the stems and leaves of rapeseed (*Brassica napus* L.). The leaves were exposed to a PEF ranging from 0.2–20 kV/cm. The study involved the analysis of rapeseed tissue to assess the extent of *Z*_p_ resulting from the application of PEF ranging from 5–20 kV/cm. Different temperatures (20–70 °C), solvent concentrations (5–100% *v*/*v* ethanol), and pH values (2–12) were examined. It was found that the most recovered polyphenols were acquired through exposure to an electric field strength of 5 kV/cm. In both stems and leaves, the optimum extraction conditions required 20 min extraction with 100% ethanol, at pH 7 and 70 °C, achieving 0.17 and 1.25 g/100 g dm, respectively. This study provides evidence to support the potential efficacy of PEF treatment as a novel approach for the valorization of rapeseed stems and leaves. This treatment selectively extracted polyphenols from the plant material, while preserving the proteins in the remaining residues.

A year later, the same research team with Yu et al. [139] investigated the impact of applying PEF before pressing rapeseed green biomass (stems) on the efficiency of polyphenol extraction. The impact of pressure, electric field strength, and pulse number on the overall polyphenol content and juice expression yield were examined. The optimal experimental conditions required electric field strength at 8 kV/cm, a total PEF time of 2 ms, with water as the solvent resulting in a significant increase in the juice expressed yield, from 34% to 81%. The press cake underwent successful dehydration, resulting in an increase in its dry matter content from 8.8% to 53.0% upon recovery. Similarly, TPC demonstrated a significant increase, surging from 0.10 to 0.48 g GAE/100 g dm after PEF pretreatment.

#### 5.3.4. Canola

Teh et al. [140] investigated four parameters, namely ethanol concentration, time, frequency, and voltage, for PEF treatment through Box–Behnken response surface methodology. These parameters were utilized to identify the most effective method for extracting polyphenols from defatted canola seed cake. PEF-assisted extraction yielded maximum results when performed at an electric field strength of 1.1 kV/cm, frequency of 30 Hz, with 10% *v*/*v* ethanol concentration for 10 μs. After PEF and MAE, the defatted canola seed cake was subjected to US. The measured responses included total phenolics, flavonoids, DPPH^•^ scavenging, and FRAP. The highest polyphenol yields (2624.18 mg GAE/100 g fw) were obtained in the optimum PEF conditions. Consequently, PEF is an economically feasible way to extract polyphenols by using low electroporation voltage which resulted in reduced solvent usage and shorter extraction time.

#### 5.3.5. Coffee and Cocoa

In order to maximize polyphenol recovery from cocoa bean shell (CBS) and coffee silver skin (CSS), the study by Barbosa-Pereira et al. [141] explored the use of PEF as a novel treatment technique. The PEF conditions were examined through response surface methodology (electric field strength 1.93–3 kV/cm, 9–16 μs pulse duration). The optimized methodology was used to analyze multiple CBS and CSS samples, after which they were separated according to their country of origin, crop type, and industrial processing. Compared to conventional extraction methods, PEF-assisted extraction resulted in roughly ~12 to 22% greater recovery yields for polyphenols (from ~26–54 mg GAE/g dw) in CBS and in ~25 to 13% recovery yields (from ~8–12 mg GAE/g dw) in CSS. Finally, the results demonstrated that the composition of bioactive compounds from various extracts of CBS and CSS, as well as their antioxidant properties, were affected by the origin, variety, and industrial processing of the raw material. These by-products of natural compounds have potential applications in agriculture, medicine, and the health sector.

#### 5.3.6. Saffron

The study from Neri et al. [142] investigated the feasibility of PEF at an electric field strength of 2 kV/cm and 1.5 kJ/kg as a pretreatment alternative to the hot air drying process to improve the quality and functional properties of saffron (*Crocus sativus* L.). TPC and antioxidant activity were measured after processing and during room-temperature aging. The application of PEF did not result in any significant alteration in TPC of fresh stigmas, averaging ~4 mg GAE/g dm. However, the highest TPC value was observed after the drying and aging process for 10 months, specifically with the application of PEF treatment and drying process (~10 mg GAE/g dm). Although the impact of PEF on the antioxidant activity of fresh stigma was found to be negative, resulting in a decrease of 24% (from an initial ~90 mmol/g dm), it was observed that the application of PEF had a positive influence on the antioxidant activity as the stigma underwent the drying process (~100 μmol/g dm). Regardless of the method of data processing, there was a significant decrease in antioxidant activity of up to 86% associated with aging after 10 months (~18 μmol/g dm). Based on the results of this investigation, it can be suggested that PEF-treated saffron exhibits promising characteristics that make it a suitable candidate for incorporation as a premium component in food applications. This is primarily attributed to the increased extraction yield, which enables the utilization of smaller quantities of the ingredients in food formulations and products. Consequently, this leads to notable cost and time savings. These concepts apply to various domains and commodities, excluding food production.

#### 5.3.7. Wheat Plants

In a study conducted by Ahmed et al. [143], an investigation was conducted to examine the impact of US and PEF on the juice derived from wheat plantlets. The treatments that involved the combination of US and PEF demonstrated elevated levels of TPC, TFC, ORAC assay, and DPPH^•^ activities in comparison to the treatments conducted separately. PEF conditions required 1 kHz of pulse frequency, 80 μs pulse width, 9 kV/cm electric field strength, 335 μs treatment time, 30 °C temperature, and 50 mL/min flow rate. The corresponding increases were 5.35% (from 305.23 μg GAE/g) for TPC, 5.51% (from 178.34 μg CE/g) for TFC, 4.91% (from 1.63 mmol TE/L) for DPPH^•^ assay, and 1.36% (from 5.12 mmol TE/L) for ORAC assay. When both treatments were employed, the increases were 8.59, 14.06, 6.74, and 2.34%, respectively.

#### 5.3.8. Sage

Sage (*Salvia officinalis* L.) leaf phytochemicals were the primary focus of the research conducted by Athanasiadis et al. [144], which aimed to evaluate the efficiency of PEF-assisted extraction. Among the variables tested was the pulse duration of PEF, which varied from 10–100 μs over the course of 30 min. They also investigated the efficacy of several “green” extraction solvents, including ethanol, water, and their respective 25–75% *v*/*v* mixtures. The obtained extracts were evaluated against those obtained without PEF as a standard of comparison. Total polyphenols, isolated polyphenols, volatile compounds, and oxidation resistance were measured to assess extraction efficacy. The highest PEF contribution to total and individual polyphenols, as well as rosmarinic acid extractability, was achieved under conditions of a 25% *v*/*v* aqueous ethanol solvent, a pulse duration of 100 μs, and an electric field strength of 1 kV/cm. The result was an increase of 73.2% in TPC (from ~24 mg GAE/g dm) and 403.1% in rosmarinic acid (from 0.37 mg/g) over the control extract. Differential scanning calorimetry was also able to confirm the results. The oxidation temperature of the PEF-treated extracts was on average 61.5% higher than that of the reference extracts (182 °C). The primary compounds, which accounted for roughly the same percentage of the total composition (65.51 and 67.58%, respectively), were ultimately detected in both the PEF-treated and the reference extracts. These findings indicated that the application of low energy intensities through PEF may result in subtle changes to the odor of the tested extracts.

#### 5.3.9. Drumstick Tree

Freeze-dried leaves of the drumstick tree (*Moringa oleifera*) were the subject of an investigation by Bozinou et al. [46]. The effectiveness of PEF extraction was compared to that of several other methods, such as MAE and UAE, as well as boiling water extraction and plain maceration (the control). The control sample was made using the same volume of freeze-dried leaves that were submerged in the same volume of double-distilled water at room temperature for 40 min. A 7 kV/cm electric field strength was required with pulse duration (PD) between 10 and 100 ms, and pulse interval (PI) between 25 and 100 μs. The PEF-treated sample with 20 ms PD and 100 μs PI achieved the highest TPC (40.24 mg GAE/g dw), at a rate that was 45% higher than that of the control sample. Other assays measuring antioxidant capacity showed the expected pattern, including % scavenging activity and ferric reducing antioxidant power (FRAP). TPC decreased in other PEF-treated samples with long PI (100 μs) and growing PD (50–100 ms). Therefore, it is important to emphasize that the optimal condition for extracting total polyphenols from freeze-dried *M. oleifera* leaves is a combination of low PD and high PI.

#### 5.3.10. Almond

Due to their high nutrient content, almonds are widely regarded as one of the world’s most valuable fruits. Additionally, in recent years, there has been a shift in focus toward almond by-products such as skins, shells, and hulls, which are abundant but underutilized. In particular, this technology was employed to create a workable valorization strategy by providing a more environmentally friendly alternative to conventional methods of polyphenols extraction. Considering this, Salgado-Ramos et al. [145] studied the innovative PEF method to valorize almond hull biomass. An electric field strength of 3 kV/cm, frequency of 2 Hz, and pulse duration of 100 ms were used to apply a specific energy of 100 kJ/kg. When compared to the traditional maceration method, PEF resulted in an extraction of Trolox equivalent antioxidant capacity (TEAC) values. In relation to the TEAC results, it was observed that the PEF-treated sample exhibited higher values in comparison to the control sample. Specifically, the PEF-treated sample displayed a value of 13.71 μM TE, while the control sample had a value of 7.78 μM TE. However, the results revealed statistically non-significant differences (*p* < 0.05). Specifically, TPC values in PEF-treated samples recorded 2.72 mg GAE/mL and were marginally higher than those of the control group 2.27 mg GAE/mL. In addition, results in ORAC revealed that PEF had a negative effect on samples, as the control sample exhibited a higher ORAC result (47.74 mM TE) compared to the PEF-treated sample (33.28 mM TE).

#### 5.3.11. Hemp

The research by Teh et al. [146] aimed to assess whether the impact of PEF treatment could improve polyphenol extraction yields from defatted hemp seed (*Cannabis sativa*) cake. A Box–Behnken design of response surface methodology was used to optimize the extraction parameters. Four independent variables, including ethanol concentration (0, 5, and 10% *v*/*v*), time (10, 20, and 30 s), frequency (30, 40, and 50 Hz), and voltage (30, 40, and 50 V), were used in a Box–Behnken design to create a model for the observed response. The 900 burst pulses, 20 μs pulse width, and 10 kJ energy were all predetermined values for the PEF process. The results showed that the optimum PEF conditions were from design point 16 which required 5% *v*/*v* ethanol concentration, 20 s treatment time, 30 Hz frequency, and 30 V. TPC ranged from 467.5–1013.0 mg GAE/100 g fw, total flavonoids from 6.36–15.13 mg luteolin equivalents (LUE)/100 g fw, DPPH^•^ % inhibition from 12.65–22.06, and FRAP from 5.54–12.22 μmol Fe^+2^/g fw. However, by incorporating the composite desirability, optimum PEF conditions required 10% *v*/*v* ethanol concentration, 10 s treatment time, 30 Hz frequency, and 30 V. The corresponding values were 1025.57 mg GAE/100 g fw, 15.76 mg LUE/100 g fw, 22.84%, and 12.75 μmol Fe^+2^/g fw.

#### 5.3.12. Sesame

There is a growing demand in the industry for extraction processes that employ reduced or zero quantities of organic solvents and operate at lower temperatures. The extraction process facilitated by PEF induces cellular damage, which subsequently enhances the diffusion of the product into the solvent. As such, a study by Sarkis et al. [147] examined the impact of PEF on the extraction of sesame cake compounds. The electric field for PEF was 13.3 kV/cm. The pulse lasted 10 μs and occurred at a rate of 0.5 Hz. By employing 10% *v*/*v* ethanol as extraction solvent, PEF recorded a TPC value of ~400 mg GAE/100 g dm, whereas the untreated sample recorded ~320 mg GAE/100 g dm. The results of this study demonstrated that polyphenol extraction using ethanol as a solvent can be reduced when the PEF method is used, as can the need for increased temperatures to enhance diffusion.

#### 5.3.13. Rice

The brown rice bran extraction with the PEF process has been studied for the first time by Quagliariello et al. [148]. PEF conditions had electric field strength of 2 kV/cm, 1000 pulses, and specific energy of 64 kJ/kg increased the brown rice’s antioxidant activity by 50% (from ~260 to ~390 μg AAE/g). In addition, several phenolic acids, such as chlorogenic acid and ferulic acid, were increased from 53.5 to 65.7 μg/g and from 16.4 to 20.9 μg/g, respectively. Therefore, it appears that including PEF pretreatment in the solvent extraction process of polyphenols from brown rice is a promising practice that will significantly increase their biological activity.

#### 5.3.14. Spruce

Bouras et al. [149] studied the effect of PEF treatment in polyphenol extraction from Nordic spruce bark (*Picea abies* L.). Norway spruce was used to isolate several polyphenols, including phenylpropanoids, tannins, flavonoids, lignans, and stilbenes. Two PEF treatment protocols at an electric field strength of 20 kV/cm and 1–400 number of pulses and pulse duration of 10 μs were tested to determine the feasibility of PEF treatment. Sodium hydroxide solution (0.01 M) was used as an extraction solvent. The results showed that samples had TPC boosted by PEF treatment. For instance, TPC has increased by over a factor of 8 (from 0.96 to 8.52 g GAE/100 g dm). The PEF treatment did not result in any visible degradation of bark tissue, suggesting it could be a viable alternative to milling that saves energy. The obtained results are promising and open up new avenues for the valorization of wood bark.

#### 5.3.15. Barberry

Barberry is a useful plant in treating various diseases, containing valuable compounds in its pruned waste. The study conducted by Sarraf et al. [150] investigated the quantity of berberine, polyphenols, and antioxidant activity present in barberry fruits, leaves, and stems of varying species, including *Berberis integerrima* and *Berberis thunbergia*. This study used a central composite design of RSM to examine the effects of variables on the extraction of berberine from the stem of *B. integerrima* (time: 2–24 h, temperature: 24–70 °C, and ethanol concentration: 50–90% *v*/*v*). Berberine concentration, DPPH^•^ scavenging capacity, and TPC were used as evaluation criteria. Additionally, pretreatment with PEF-assisted was used prior to extraction with electric field strengths of 250, 1000, and 1250 V/cm, pulse numbers of 50 and 100, and frequency of 1 Hz. The berberine concentration with PEF treatment rose dramatically. The stem of *B. integrrima* was chosen for further study because it had the highest levels of antioxidant activity and berberine content. The ideal maceration conditions were 90% ethanol, 70 °C, and 3.36 h (141.6 min). Among the various techniques, maceration with 100 pulses and a field strength of 1 kV/cm was the most effective. Berberine concentration was 1.86 mg/g, TPC was measured at 11.11 mg GAE/g, and antioxidant activity was 71.84% in the optimal maceration condition. The PEF-assisted method increased berberine content to 2.78 mg/g, TPC to 14.57 mg GAE/g, and antioxidant activity to 78.6%, respectively. The results showed that the stem extract from *B. integrrima* is rich in berberine and antioxidants and could be used in a number of different sectors.

#### 5.3.16. Other Plants

Extracts from medicinal and aromatic plants are widely used for their health benefits to humans. However, it is difficult because the success of the process of extracting polyphenols from plants is highly dependent on the technique used and the operating conditions that are imposed. Ziagova et al. [97] investigated the impact of PEF on several plants and herbs. By implementing an electric field strength of 0.5–2 kV/cm, they measured TPC (mg GAE/g dw) in several samples, such as *Melissa officinalis* L. leaves (155), *Cistus incanus* L. *creticus* leaves (148), and *Aronia melanocarpa* L. fruit (67), and *Crocus sativus* L. petals (147). High extraction yields and the biological stability of bioactive compounds are achieved using the combined process of PEF and US proposed in this study. It has advantages compared to the conventional or the most advanced extraction methods due to the application of short extraction time moderate temperatures and the use of water as the solvent.

Extracting polyphenols from the plants *Rosa canina*, *Calendula officinalis*, and *Castanea sativa* using PEF treatment was the objective of the study by Lakka et al. [151]. Traditionally, these plants have been used not only to make medicinal decoctions but also to add flavor to drinks of all kinds. Electric field strength was applied at intensities between 1.2–2.0 kV/cm in pulses of 10 μs duration. The samples were extracted for 20 min, during which time the PEF period was set to 1 ms. In order to track and assess the extracts, their TPC and individual polyphenolic compounds were calculated in comparison to untreated samples. The PEF process appeared to increase polyphenols extraction from all three plant materials tested. TPC in *R. canina* fruits increased to 63.79% (from ~42 mg GAE/g dw) and eriodictyol-7-*O*-rutinoside recovery increased to 84% (from 0.032 mg/g dw) when 1.4 kV/cm was employed, respectively. Regarding *C. officinalis* the corresponding TPC and isorhamnetin-3-*O*-rutinoside increase were 55.02% (from ~35 mg GAE/g dw) and 73% (from 7.868 mg/g dw) through electric field strength of 1.2 kV/cm. Finally, by employing the same electric field strength (1.2 kV/cm) *C. sativa*, the TPC increase was 48.41% (from ~115 mg GAE/g dw), whereas quercetin 3-*O*-glucoside was increased by 82% (from 1.153 mg/g dw). Understanding the potential of PEF will allow the development of more potent extracts that can be used to fortify medicinal herbal teas, traditional beverages, and even alcoholic beverages.

This study by Carpentieri et al. [152] compared the efficiency of hydroethanolic extraction (0–50% *v*/*v* ethanol in water) for up to 4 h following PEF treatment or US on the cell disintegration of two Mediterranean herb tissues (*Origanum vulgare* L., *Thymus serpyllum* L.). The extraction rate of polyphenols decreased over time, as predicted by Peleg’s model (R^2^ = 0.898–0.989). When applied before solid-liquid extraction, either PEF or US treatment had the potential to shorten the extraction time and lower the ethanol concentration required to recover the same amount of phenolic compounds. Increased values in TPC of *O. vulgare* (36% from ~100 mg GAE/g dw) and *T. serpyllum* (36% from ~40 mg GAE/g dw). The corresponding values in antioxidant activity (FRAP) were also found to increase (29% from 103.9 μmol Fe^+2^/g dw) and (47% from 31.1 μmol Fe^+2^/g dw) of extracts obtained from PEF-pretreated herb samples under optimum PEF conditions (3 kV/cm, 10 kJ/kg). No measurable degradation of individual polyphenols from PEF treatment, as determined by GC-MS analysis.

#### 5.3.17. Algae/Microalgae

Microalgae contain polyphenols and coloring compounds that exhibit antioxidant, antibacterial, and anti-inflammatory properties [153,154]. It is worth mentioning that the PEF system was even applied to four species of these algae. To investigate the effects of various treatments on the brown alga *Laminaria digitata*, crude aqueous extracts were prepared using PEF-assisted extraction in a study by Einarsdóttir et al. [155]. Biomass concentration (0.17–3.28% dw), number of pulses during PEF treatment (12–268 pulses), and initial temperature of algae suspension (12–48 °C) were the three factors used for response surface methodology. An electric field of 7.5 kV/cm and a frequency of 1.2 Hz were applied to the samples. The extraction yield was 15%, and the supernatant yield was 70%. TPC was measured at 4 mg GAE/100 g dw. The lowest biomass concentration also had the highest supernatant yield, polyphenol content, and carbohydrate content. A positive relationship between the temperature rises and the total number of PEF pulses was observed. This research demonstrates that valuable compounds in *L. digitata* can be extracted using PEF-assisted extraction rather than extreme temperatures or organic solvents.

Recovery of polyphenols from the microalgae *Tetraselmis chuii* and *Phaeodactylum tricornutum* was tested using PEF-assisted extraction in combination with aqueous or dimethyl sulfoxide (DMSO) solvents. The study by Kokkali et al. [156] investigated several PEF parameters. The specific energy input was 100 kJ/kg, whereas two PEF treatments were administered (1 kV/cm with 400 pulses and 3 kV/cm with 45 pulses). PEF treatment at 3 kV/cm and 4 h of extraction yielded the highest value regardless of the solvent (water yielded 6.42 GAE/g dw, and DMSO 50% in water yielded 6.70 mg GAE/g dw) for *T. chuii*, DMSO was only effective at enhancing polyphenol extraction from *P. tricornutum*. In *P. tricornutum*, PEF pretreatment with DMSO 50% in water as extraction solvent resulted in the highest extraction yield, with values of ~8 mg GAE/g dw. Finally, PEF shows promise as a potential tool for improving the selective extraction of antioxidant bioactive compounds from microalgae. 

Research conducted by Castejón et al. [157] presented three Icelandic species of algae, *Ulva lactuca*, *Alaria esculenta,* and *Palmaria palmata,* and the effect of extraction with a heated water bath, PEF, and the combination of these methods. The PEF conditions required an electric field strength of 8 kV/cm at 1.2 Hz for 10 min with 3 pulses. Several advantages of PEF were revealed, including its non-thermal nature and shorter extraction time (10 vs 45 min), and PEF showed results that were comparable to the conventional method. However, the PEF-treated *Alaria esculenta* sample had the highest TPC of 9.37 mg GAE/g dw when compared to the heat water process (+4.8%) and TFC of 12.43 mg QE/g dw, along with the greatest antioxidant capacities. PEF had a negative impact on *P. palmata*, as PEF declined TPC (1.8 mg GAE/g dw, –2.43%) but increased TFC (0.94 mg QE/g dw, +16%). In *U. lactuca*, PEF had a mostly deleterious effect in both TPC (1.59 mg GAE/g dw, –22%) and TFC (3.43 mg QE/g dw, –32%). Therefore, this exploratory research indicates that PEF-produced extracts of Icelandic *Alaria esculenta* may be useful as ingredients in natural cosmetic and cosmeceutical formulations. However, the deleterious impact of PEF should be further investigated.

The last microalgae studied was *Spirulina*, which is listed by the European Commission as a new type of food for daily nutrition. Three different extraction methods were studied by Zhou et al. [158] for the *Spirulina* samples, namely PEF, pressurized liquid extraction (PLE), and a combination of PEF and PLE, with water as extraction solvent. The PEF condition required an electric field strength of 3 kV/cm, 44 pulses, specific energy 99 kJ/kg. In comparison to the conventional control technique of Folch extraction, the combination of PEF and PLE resulted in a significant reduction in extraction time (~165 min) and a significant increase (*p* < 0.05) in TPC values of *Spirulina* extracts, from ~2 to ~20 mg/g dw. Table 3 provides a summary of the above studies on various plants, herbs, nuts, and seaweeds.

## 6. Current Challenges and Limitations

PEF technology has many applications in waste valorization, but its widespread adoption has been hampered by a number of challenges [159]. The initial cost of the PEF system is the major issue for PEF-assisted waste valorization [160]. Treatment chamber electrodes undergo electrochemical changes, so durable and low-cost electrodes are required as well. In addition, food waste mainly exists in solid, semi-solid, and liquid states, so the PEF treatment chamber should be redesigned to maximize extraction efficiency [161]. The utilization of PEF in the context of solid waste is currently in its early stages. Due to the variability in electrical resistivity observed in solid waste, and the outcomes of PEF treatment can be less repeatable, leading to instances of untreated regions alongside areas that may be subjected to excessive treatment [162].

Utilizing food waste is crucial for the sustainability of food industry [163]. At the moment, the food industry is mainly concerned with recycling waste and reducing energy and water consumption [164]. A huge challenge would be a large-scale utilization of these several food by-products for increased collaboration between academia and industry. The application of PEF technique for the recovery of compounds from these wastes could create a high value-added product despite its relatively low market value [165,166]. According to the available literature, more research is needed into the effectiveness of PEF pre-treatment for food wastes before it can be used consistently in industrial scale [49].

The majority of scientific investigations have been carried out using laboratory-scale apparatus, employing small sample volumes and batch flow configurations. Consequently, engineering difficulties exist in scaling up PEF processes for fresh fruits, vegetables, and their corresponding byproducts. Due to the need to consider variations in treatment uniformity, and residence times, the use of such data to pilot-scale or full commercial-scale production is frequently unfeasible [167]. Nevertheless, there has been significant progress in the development of PEF equipment specifically designed for industrial applications. This equipment has demonstrated successful implementation in Europe, where it has been utilized to effectively enhance the shelf life of fruit juices at a remarkable rate of up to 8000 L/h [168]. According to Toepfl et al. [169], the estimated cost range for processing PEF is between 0.02–0.03 USD/L.

Finally, extraction yield, extraction time, and specific energy consumption are three parameters that have been compared across studies of modern pretreatment PEF methods [170,171]. However, several cutting-edge techniques could be combined with PEF in order to enhance polyphenol extraction, therefore they need further investigation. Although, it is of high importance to emphasize that the combination of PEF and US led to deleterious effects on the final product, since less polyphenols were measured in a sample where both methods were performed than in a control sample. The quality standards of the final product should be taken into account when deciding which pretreatment method to use [172].

## 7. Conclusions and Future Perspectives

Numerous studies have been conducted to investigate the impact of applying PEF on the polyphenol extraction of different samples. The application of PEF extends to a variety of agricultural products, including fruits, vegetables, aromatic and medicinal plants, as well as wine and algae. In the majority of instances, the utilization of PEF-assisted extraction resulted in a substantial increase in the extraction of polyphenols in the examined sample. Also, the lack of evidence supporting the view that PEF is a destructive technique for the specific bioactive compounds under investigation is of high importance. This finding suggests that the adoption of PEF is not only environmentally and economically sustainable, but also provides access to a variety of food options that are abundant in antioxidant compounds. 

The PEF pretreatment technique still presents several obstacles that necessitate future resolution. Firstly, extraction kinetics models should be developed and tested, and the mechanisms by which PEF is extracted should be confirmed. Future practical applications will necessitate further research into the mechanism and the creation of a scientific model of mass transfer. Also, PEF applications in the food industry offer a chance to implement large-scale energy-efficient processes that would result in minimally processed products with a higher concentration of bioactive compounds. Despite PEF being a non-destructive method for bioactive compounds, a deleterious effect of PEF with other pretreatment or extraction methods was observed, thus, a thorough investigation of the reason behind that incidence is of high importance. Juice production has been significantly enhanced through the use of PEF on fruits. However, it should be noted that the release of enzymes has caused a general decline in the quality of fruit juices as a result of PEF usage. Further investigation is also needed to determine if and how these treatments can be used to produce safe and stable products that retain their fresh-like bioactive potential. In addition, PEF technology can be used to extract bioactive compounds from wastewater discharged from the dairy, meat, and seafood processing industries, allowing these businesses to maximize the value of this water resource with minimal additional expense. Society and the scientific community would benefit from optimizing PEF treatment conditions for extracting bioactive compounds from the above residues. To fully accomplish the objectives above, it is imperative that the PEF technique be implemented on a large-scale within the industrial sector. Finally, it is crucial that authors provide all relevant experimental details to enable replication and comparison of results.

## Figures and Tables

**Table 1:** Application of PEF on fresh fruit and by-products with the treatment effects.

Sample	PEF Conditions	Treatment Effect	Ref.
Apricot	1 kV/cm, pulse frequency 1000 μs, 10 μs pulse duration	Increases by 88% in TPC (from ~3.5 to ~6.5 mg GAE/g dw) and 100% in TFC (from 3.78 to ~7.5 mg RtE/g dw)	[77]
Blackthorn	1.0 kV/cm, 1 ms pulse period, 10 μs pulse length	Increased TPC value by 27% (from 24.20 to 30.74 mg GAE/g) when compared to stirring,	[78]
Cherry	2.5 kV/cm, 20 μs, 100 Hz, pulse number 385–10,000	Rutin concentration increased by 54% (from 5.04 to 7.77 μg/g ww)	[83]
Grape	5 kV/cm, 1 ms pulse duration, 42–53 kJ/kg	Increase in anthocyanin content by 62% (from 186 to ~301 mg/L)	[84]
	0.7 kV/cm, 200 ms treatment duration	~19% increase in anthocyanins (from ~480 to ~570 mg/L), 36% increase in tannins (from 2.5 to 3.4 mg/L)	[86]
	0.7 kV/cm, 40 ms treatment duration	~10% increase in TPC (from~870 to ~970 mg/L), ~18% increase in tannins (from ~2.7 to ~3.2 g/L)	[87]
	8 kV/cm, 6.7 kJ/kg, 45 μs pulse duration	TPI increased by ~19% from 61.15 to 73.15	[88]
	4 kV/cm, 3.7 pulses of 100 μs width, 6.2 kJ/kg	High values of TPI (~60 AU compared to ~45 AU from untreated samples), anthocyanins (from ~480 to ~500 mg/L), and tannins (from ~1 to ~1.5 g/L) were achieved	[89]
	1.5 kV/cm, 10 μs pulse length, 20 kJ/kg, 250 L/h	TPI: 41.3% increase (from 26.3 to 44.8), anthocyanins: 50% increase (from 39 to 78 mg/L), tannins 50% increase (from 1.2 to 2.4 g/L)	[90]
	1.4 kV/cm, 10 μs pulse duration, 1 ms treatment time	Increases in TPC from to ~56 to ~110 mg GAE/g dw (49.15%), Quercetin-3-rutinoside from 0.012 to 0.083 mg/g dw (85%), Kaempferol-3-glucoside from 0.052 to 0.153 mg/g dw (66%), Gallic acid from 0.045 to 0.0124 mg/g dw (63%)	[91]
	0.9–3 kV/cm, 10.4–32.5 kJ/kg	TPC increased by ~55% (from 197 to 439 mg GAE/L)	[96]
Grape juice	5 kV/cm, 63.4 kJ/kg, 40 μs pulse width	TPC increased by ~56% (from 916 to 1434 mg GAE/L)	[95]
Wine	5 kV/cm, 1 ms treatment duration, 48 kJ/kg	TPC increased by 17–178% (from 130.9 and 305 to 364.1 and 359.8 mg/L)	[85]
Grape stem	1 kV/cm, treatment duration 30 min	PEF only: 4% increased TPC (from 0.048 to 0.05 AU)	[98]
Grape leaf	0.5–2 kV/cm	High TPC value (97 mg GAE/g dw)	[97]
Grape pomace/seed	1.2 kV/cm, 18 kJ/kg	Increases in gallic acid from 4.53 to 7.40 mg/100 g (63%) and TPC from 60.98 to 113.58 mg/100 g (86%) when increasing temperature from 20 to 50 °C	[92]
	13.3 kV/cm, 0.5 Hz	At *Z*_p_ 0.8, PEF (63.47 mg/L) achieved greater anthocyanin recovery than HVED (40.64 mg/L)	[93]
	0.86 kV/cm, 13 Hz, pulse duration 900 μs, 75 ms pulse interval, 810 ms treatment time	Comparable TPC (~24 mg GAE/g) with the control sample, which was extracted with 75% ethanol, whereas the PEF-treated sample was extracted with 20% ethanol	[94]
	4.6 kV/cm, 20 kJ/kg	Increases in TPC from 8.30 to 9.51 mg GAE/g dm (15%), TFC from 36.68 to 58.53 mg QE/g dm (60%), TAC from 0.84 to 1.03 mg C3G/g dm (23%), and in TC from 3.84 to 5.45 mg TC/g dm	[99]
	0.5–2 kV/cm	High TPC value (31 mg GAE/g dw)	[97]
Apple tissue	3 kV/cm, 100 pulses	TPC increased by ~10% from 426.69 to 472.05 mg chlorogenic acid/100 g dm	[101]
Apple juice	30 kV/cm	Non-significant differences in TPC (from 337.51 to 340.70 mg/L), reduction in AA from 17.40 to 16.74 μmol Trolox/mL)	[102]
Apple pomace	30 kV/cm, 17 kJ/kg or20 kV/cm, 100 kJ/kg	TPC: the lowest concentration (220 μg GAE/g) when PEF with 30% *v*/*v* EtOH was used as extraction solvent compared to UAE (800 μg GAE/g), and ASE (~420 μg GAE/g)	[103]
Apple	1 kV/cm, 20 Hz pulse frequency, and 7 μs pulse width	Dry the sample efficiently, TPC measured 1257 mg GAE/100 dm	[104]
Pomegranate peel	10 kV/cm, 90–100 kJ/kg	TPC through PEF measured at 39.2 mg GAE/g dm, ~15% lower by HVED, ~169% higher than the US, ~388% higher than IR, ~680% higher than water bath treatment	[107]
	0.5–2 kV/cm	High TPC value (208 mg GAE/g dw)	[97]
Citrus juice	3 kV/cm	TPC increased by ~49%(orange) from ~36 to ~70 mg/100 mL, ~50% (lemon) from ~30 to ~60 mg/100 mL, ~60% (pomelo) from ~32 to ~80 mg/100 mL	[109]
Citrus peel	10 kV/cm	Increase in major polyphenols in orange (hesperidin, ~5%) from 4.85 to 5.07 mg/g dm, pomelo (naringin, ~41%) from 7.35 to 10.36 mg/g dm, a decrease in major polyphenol of lemon (eriocitrin, ~112%) from 3.06 to 1.44 mg/g dm	
Orange peel	1 kV/cm, 10 μs pulse duration, 1 ms treatment period	TPC increase by 25% (from 27.70 to 34.71 mg GAE/g dw) and hesperidin content by 19% (from 13.67 to 16.26 mg/g dw)	[110]
	1–7 kV/cm, 5–50 pulses of 3 s each	Increased concentrations of naringin from 1 to 3.1 mg/100 g fw (210%), hesperidin from 1.3 to 4.6 mg/100 g fw (253%)	[111]
Lemon peel	7 kV/cm, 90 μs pulse duration	TPC increased by 150% from ~64 to 160 mg GAE/100 g dw, eriocitrin concentration from 30.39 to 176.35 mg/100 g fw, and hesperidin concentration from 15.90 to 84.44 mg/100 g dw both increased by above 400%	[112]
	1.0 kV/cm, 1 ms pulse period, 10 μs pulse length	Negative impact in TPC (277% decrease) compared to conventional extraction from 51.24 to 13.56 mg GAE/g	[113]
Quince peel	1 kV/cm, 1000 Hz, 10 μs pulse duration, 1 ms pulse period	Initial increase through RSM in TPC by 8% (from 32.78 to 35.43 mg GAE/g dw), and a further increase by 34% through the PLS model as TPC reached 43.99 mg GAE/g dw	[114]
Blueberry pomace	20 kV/cm, 41.03 kJ/kg, 100 pulses	Higher values of TPC (10.52 mg GAE/g dw) than HVED (~5 mg GAE/g dw) and US methods (~6 mg GAE/g dw)	[115]
Red raspberry puree	25 kV/cm, 300 mL/min	Non-significant impact on TFC (~150 μg/mL), but increased ~16% total anthocyanin content (from ~125 to ~145 mg/L) and ~9% TPC (from ~430 to ~470 mg/L)	[116]
Blueberry puree		Non-significant impact on TFC (~310 μg/mL), increased ~15% total anthocyanin content (from ~650 to ~750 mg/L) but decreased ~6% TPC (from ~520 μg/mL)	
Cranberrybush puree	3 kV/cm, 2 Hz, 20 μs pulse width	TPC increased by ~4–14% (from initially ~400 mg GAE/100 g fw), CUPRAC antioxidant activity by ~7% (from 1500 mg TE/100 g fw)	[117]
Blackcurrants	1318 V/cm, 315 pulses	19% increase in TPC (from 3.18 mg GAE/g extract), 45% increase in AA (from 1.12 mg GAE/g extract), and 6% increase in monomeric anthocyanins content (from 1.30 mg cyanidin-3-glucoside/g extract)	[118]
Strawberry puree and juice (kale)	11.9 kV/cm, 120 kJ/kg, 20 μs pulse width	Increase in anthocyanins content from almost ~32 to 35 mg pelargonidin-3-glucoside/L in kale mix by 9%, and from 40 to 45 mg pelargonidin-3-glucoside/L (PEF-treated) in the strawberry puree by 12.5%	[119]
Tomato juice	MIPEF: 1 kV/cm, 0.1 Hz, 16 pulses of 4 μs HIPEF: 35 kV/cm, 100 Hz, 4 μs pulses	MIPEF: TPC increased by 25% from ~148 to ~180 μg/g fw, HIPEF: TPC increased by 5% from ~148 to ~155 μg/g fw	[120]
Tomato fruit	1.2 kV/cm, 30 pulses	TPC increased by 44%, as it had 144.61% relative TPC	[121]
Red prickly pear fruit	1200 V/cm, 11.44 kJ/kg, 10 Hz	PEF-treated samples increased in juice yield by 3.3 (from 16.69%) and betalain extraction by 1.48 (from 19.5 mg/100 g) compared to untreated samples	[122]

**Table 2:** Application of PEF on fresh vegetables and by-products with the treatment effects.

Sample	PEF Conditions	Treatment Effect	Ref.
Potato peel	5 kV/cm, 10 kJ/kg	Increased TPC by ~10% (from ~1160 to 1295 mg GAE/kg fw)	[125]
Asparagus root	1.6 kV/cm, 200 Hz, 20 μs pulse width	Increased values of extraction yield from 47.7 to 58.8% (23%), TPC from 32.6 to 34.4 mg GAE/g extract (5%), TFC from 0.16 to 0.17 mg RE/g extract (6%), and FRAP from 1363 to 1418 mM FeSO_4_ E/g extract (4%)	[126]
Mushrooms	38.4 kV/cm, 272 μs duration	Estimated ~26% or 1.6 mg GAE/g higher polyphenol extraction yield	[129]
Olive	0.5–2 kV/cm	High TPC value (12 mg GAE/g dw)	[97]
Olive pomace	3 kV/cm, 15 μs pulse width	Notable increase in TPC (91.6%) from ~1500 to ~2900 mg/L	[132]
Olive paste	1.5 kV/cm, 100 pulses	Increased recovery yield to 25.4% (by ~3%), TPC (by ~7%) from ~760 mg GAE/Kg oil	[133]
Olive leaf	1 kV/cm, 10 ns pulse duration	Increased TPC (by 31.85%) from 15.74 to 20.75 mg GAE/g dw	[134]
	0.85 kV/cm, 100 μs pulse period, 2 μs pulse duration	TPC increase by 38.5% (from 18.30 to 25.35 mg GAE/g dw)	[135]
	0.5–2 kV/cm	High TPC value (105 mg GAE/g dw)	[97]

**Table 3:** Application of PEF on various plants, herbs, nuts, seaweeds, and by-products with the treatment effects.

Sample	PEF Conditions	Treatment Effect	Ref.
Borage leaf	0–5 kV/cm, 10–60 min treatment duration	TPC: 1.3–6.6 times increase (from 0.3 mg GAE/g fw), ORAC: 2.0–13.7 times increase (from ~10 mg TE/g fw)	[136]
Rapeseed stem	5 kV/cm	High TPC value (0.17 g/100 g dm)	[138]
Rapeseed leaf		High TPC value (0.25 g/100 g dm)	
Rapeseed stem	8 kV/cm, 2 ms treatment duration	TPC increased by 380% (from 0.10 to 0.48 g GAE/100 g dm)	[139]
Canola seed cake	1.1 kV/cm, 30 Hz, 10 s exposure time	High TPC (2624.18 mg GAE/100 g fw) yielded in a short time	[140]
Cocoa bean shell	1.93–3 kV/cm, 9–16 μs pulse duration	Up to 22% increase in TPC (from ~26–54 mg GAE/g dw)	[141]
Coffee silver skin		Up to 13% increase in TPC (from ~8–12 mg GAE/g dw)	
Saffron	2 kV/cm, 1.5 kJ/kg	Non-significant increase in TPC compared to untreated samples (~4 mg GAE/g dm), significant decrease in AA to ~18 μmol/g dm (~86%) when aging after 10 months	[142]
*T. chuii*	3 kV/cm, 45 pulses, 100 kJ/kg	High TPC yield (~6.7 mg GAE/g dw)	[156]
*P. tricornutum*	1 kV/cm, 400 pulses, 100 kJ/kg	High TPC yield (~8 mg GAE/g dw)	
Wheat plantlet	9 kV/cm, 1 kHz, 80 μs pulse width, 335 μs treatment time	Increase in TPC from 305.23 μg GAE/g (5.35%), in TFC from 178.34 μg CE/g (5.51%), in DPPH from 1.63 mmol TE/L (4.91%), and in ORAC from 5.12 mmol TE/L (1.36%)	[143]
Sage leaf	1 kV/cm, 100 μs pulse duration	Increase in TPC by 73.2% from ~24 mg GAE/g dm) and in rosmarinic acid concentration by 403.1% from 0.37 mg/g	[144]
Almond hull	3 kV/cm, 2 Hz, 100 kJ/kg, 100 ms pulse duration	Slight increase in TPC (~19%) from 2.27 to 2.72 mg GAE/mL	[145]
Hemp seed	30 V, 30 Hz, 10 s treatment time	High TPC (1025.57 mg GAE/100 g fw) and TFC (15.76 mg LUE/100 g fw)	[146]
Sesame cake	13.3 kV/cm, 0.5 Hz, 10 μs	TPC increased by ~25% from ~320 to ~400 mg GAE/100 g dm	[147]
Rice	2 kV/cm, 64 kJ/kg, 1000 pulses	TPC increased by ~50% from ~260 to ~390 μg AAE/g	[148]
Spruce bark	20 kV/cm, 10 μs pulse duration, 1–400 pulses	TPC increased 8 times (from 0.96 to 8.52 g GAE/100 g dm)	[149]
Barberry	1.0 kV/cm, 100 pulses	Increase in TPC by 30% (from 11.11 to 14.57 mg GAE/g) and berberine content by 49% (from 1.86 to 2.78 mg/g)	[150]
*R. canina*	1.4 kV/cm, 10 μs pulse duration	Increase in TPC by 63.79% (from ~42 mg GAE/g dw) and in eriodictyol-7-*O*-rutinoside concentration by 84% (from 0.032 mg/g dw)	[151]
*C. officinalis*	1.2 kV/cm, 10 μs pulse duration	Increase in TPC by55.02% (from ~35 mg GAE/g dw) and in isorhamnetin-3-*O*-rutinoside concentration by 73% (from 7.868 mg/g dw)	
*C. sativa*		Increase in TPC by 48.41% (from ~115 mg GAE/g dw) and isorhamnetin-3-*O*-rutinoside concentration by 82% (from 1.153 mg/g dw)	
*L* *. digitata*	7.5 kV/cm, 1.2 Hz	High extraction yield (15%), supernatant yield (70%), TPC (4 mg GAE/100 g dw)	[155]
*O. vulgare*	3 kV/cm, 10 kJ/kg	Increase in TPC by 36% from ~100 mg GAE/g dw and in FRAP by 29% from 103.9 μmol Fe^+2^/g dw	[152]
*T. serpyllum*		Increase in TPC by 36% from ~40 mg GAE/g dw and in FRAP by 47% from 31.1 μmol Fe^+2^/g dw	
*M. officinalis* L. leaf	0.5–2 kV/cm	High TPC value (155 mg GAE/g dw)	[97]
*C. incanus* L. spp. *creticus* leaf		High TPC value (148 mg GAE/g dw)	
*C. sativus* L. petal		High TPC value (147 mg GAE/g dw)	
*A. melanocarpa* L. fruit		High TPC value (67 mg GAE/g dw)	
Mixture of *C. sativus* L. petal and *V. vinifera* L. cv. *Xinomavro* fruit		High TPC value (54 mg GAE/g dw)	
Flaxseed hull	20 kV/cm, treatment duration 10 ms, 300 kJ/kg.	PEF: High TPC (1000 mg GAE/100 g) with alkaline hydrolysis compared to acidic hydrolysis (270 mg GAE/100 g dm)	[137]
Drumstick tree leaves	7 kV/cm, 20 ms pulse duration, 100 μs pulse interval	Increased TPC by ~45%, achieving 40.24 mg GAE/g dw	[46]
*A. esculenta*	8 kV/cm, 1.2 Hz, 10 min treatment duration, 3 pulses	Both TPC and TFC were slightly increased (by ~5% and ~1.5%) from 8.94 mg GAE/g dw and 12.23 mg QE/g dw, respectively	[157]
*P. palmata*		TPC after PEF decreased by 2.43% (1.8 mg GAE/g dw), TFC increased by 16% (0.94 mg QE/g dw)	
*U. lactuca*		Both TPC (1.59 mg GAE/g dw) and TFC (3.43 mg QE/g dw) dramatically decreased after PEF treatment (by –22% and –32%, respectively)	
*Spirulina*	3 kV/cm, 99 kJ/kg, 44 pulses	Significant TPC increase (by ~900%) from ~2 to ~20 mg/g dw	[158]

## Data Availability

Not applicable.
